# The effect of long-term spaceflight on drug potency and the risk of medication failure

**DOI:** 10.1038/s41526-023-00271-6

**Published:** 2023-05-05

**Authors:** J. F. Reichard, S. E. Phelps, K. R. Lehnhardt, M. Young, B. D. Easter

**Affiliations:** 1 NASA Johnson Space Center, Houston, TX US; 2 Department of Environmental and Public Health Sciences, University of Cincinnati, Cincinnati, OH USA; 3 KBR, Houston, TX USA; 4 Department of Public Health & Preventive Medicine, University of Texas Medical Branch, Galveston, TX USA; 5 Departments of Emergency Medicine & Neuroscience, Emory University, Atlanta, GA USA; 6 Department of Emergency Medicine and Center for Space Medicine, Baylor College of Medicine, Houston, TX USA; 7 Department of Emergency Medicine, University of Colorado School of Medicine, Denver, CO USA

**Keywords:** Drug discovery and development, Medical research, Health care

## Abstract

Pharmaceuticals selected for exploration space missions must remain stable and effective throughout mission timeframes. Although there have been six spaceflight drug stability studies, there has not been a comprehensive analytical analysis of these data. We sought to use these studies to quantify the rate of spaceflight drug degradation and the time-dependent probability of drug failure resulting from the loss of active pharmaceutical ingredient (API). Additionally, existing spaceflight drug stability studies were reviewed to identify research gaps to be addressed prior to exploration missions. Data were extracted from the six spaceflight studies to quantify API loss for 36 drug products with long-duration exposure to spaceflight. Medications stored for up to 2.4 years in low Earth orbit (LEO) exhibit a small increase in the rate of API loss with a corresponding increase in risk of product failure. Overall, the potency for all spaceflight-exposed medications remains within 10% of terrestrial lot-matched control with a ~1.5 increase in degradation rate. All existing studies of spaceflight drug stability have focused primarily on repackaged solid oral medications, which is important because non-protective repackaging is a well-established factor contributing to loss of drug potency. The factor most detrimental to drug stability appears to be nonprotective drug repackaging, based on premature failure of drug products in the terrestrial control group. The result of this study supports a critical need to evaluate the effects of current repackaging processes on drug shelf life, and to develop and *validate* suitable protective repackaging strategies that help assure the stability of medications throughout the full duration of exploration space missions.

## Introduction

As the National Aeronautics and Space Administration (NASA) and its international partners seek to develop capabilities to conduct exploration space missions away from Earth’s orbit, it is anticipated that roundtrip planetary missions to Mars will exceed two years in duration^1^. Unlike regular resupply to the International Space Station (ISS), planetary missions will be too distant for resupply and therefore need to be self-sufficient. Long-duration exploration missions will expose astronauts to new and increased hazards and, therefore, an anticipated higher incidence of medical conditions compared to current mission scenarios. At the same time, resource constraints on exploration spacecraft will require that those vehicle systems (including the medical system) function with less mass/volume/power^2^. Pharmaceuticals are a critical resource required to manage high-probability or potentially severe medical conditions during deep space missions. Therefore, pharmaceuticals selected for exploration missions must remain stable throughout the entire timeframe of such missions^3^.

Drug products undergo degradation over time. Drug degradation is a chemical reaction that typically progresses at a consistent rate under a consistent set of storage conditions, assuming co-reactants are available in excess^4–7^. Many active pharmaceutical ingredients (APIs) are susceptible to degradation when exposed to atmospheric factors (e.g., oxygen or humidity), non-ionizing radiation (e.g., ultraviolet light) or ionizing radiation (e.g., gamma and alpha radiation). Degradation of finished drug products can be complex and, in addition to environmental factors, may also involve interactions of the API with excipients, which are pharmacologically inactive but not chemically inert. Chemical degradation in a drug product can result in both the loss of the API (i.e., loss of potency) and/or accumulation of impurities^5^. Degradation can also result in physical changes, which for solid oral drugs may include changes in moisture content, color, and hardness, and for nonsolid medications, phase separation, and other changes depending on the formulation. As a step toward characterizing the relative effect(s) of spaceflight on drug stability, we focus on the loss of potency associated with long-term spaceflight. Degradation impurities are also an important source of uncertainty that requires investigation in future studies, however available spaceflight studies do not permit relative quantitation of impurities in spaceflight and terrestrial medications. The Shelf Life Extension Program (SLEP) reported that of the 59 drug products with initial extension failures, 35 products failed based on potency (assay) criteria, compared to only seven that failed based on impurity or degradant content^8^. For this reason, quantitative evaluation of drug potency based on stability-indicating methods is a reasonable step towards characterizing spaceflight stability of medications.

NASA has previously supported six investigations into the stability of drugs after prolonged storage in LEO on board the International Space Station (ISS)^9–14^. With the exception of the study by Du et al.^14^, all these studies (5/6) have been opportunistic designs that take advantage of sui generis medications returned from orbit after varying periods of spaceflight. None of the studies include controls for comparative evaluation of LEO spaceflight drug stability. In contrast, Du et al. conducted a longitudinal drug stability study where spaceflight drugs were matched to corresponding terrestrial controls from the same manufacturing lot across four time points^14^. Despite the advantages of this study design, the Du et al.^14^ study is limited because it provides only a qualitative analysis of stability for an arbitrarily selected subset of the tested drugs rather than a quantitative assessment of overall drug stability and time-dependent failure. Consequently, uncertainty persists about the effect of spaceflight on medication stability. In this paper, we reanalyze the primary data from six previous spaceflight drug stability studies to quantify and better understand the effect of spaceflight exposure on drug potency. The goal of this work is to identify and address critical research gaps and uncertainties by implementing an experimental pharmaceutical testing strategy based on well-designed stability and stress studies.

## Results

### Relevant studies

Six primary English-language research studies were identified that reported spaceflight drug stability data. Of these studies, only two have published results. Among these studies, Du et al.^14^ performed the only study with lot-matched controls, while the five other studies used opportunistic spaceflight samples and manufacturer-matched drugs from different manufacturing lots as comparators (see Table 3). Among these studies, only one is published^12^. The four remaining investigations are non-peer reviewed NASA reports^9–11^ of which one study reanalyzed three medications that were the same samples initially tested and reported by Du et al. approximately three years earlier^13^. All available spaceflight drug stability studies are limited to LEO missions, which has significantly lower levels of ionizing radiation than are anticipated for exploration missions to the moon and beyond.

### Lot-matched controlled spaceflight study

A brief discussion of the Du et al.^14^ study is relevant to contextualize the analysis that follows. Du et al. evaluated eight medication kits, each consisting of thirty-three drug products containing 39 APIs. Of these 39 APIs, 36 were assayed for API content (API content for two drug products was not reported). Two APIs were present in more than one formulation (ciprofloxacin [3] and promethazine [3]), and three products were combination products containing two separate APIs (a fourth combination drug product containing two APIs, noted above, did not have reported assay results). Half the medication kits (4) were stored aboard ISS, and the remaining were stored terrestrially in an environmentally-controlled chamber at the Johnson Space Center (JSC) under temperature and humidity conditions similar to the flight samples. Across all of the medication kits, each drug product was from a single manufacturing lot (i.e., one lot per drug product); hence, all spaceflight drug samples were matched to control samples from the same manufacturing lot across all kits and time points. Among the solid dosage forms, 22 of the 24 drugs were removed from the original manufacturer’s container and repackaged in ridged polypropylene containers. These medication containers are not considered protective since atmospheric factors can permeate plastic containers at defined rates^15^, and there is no record that the containers were sealed or met US Pharmacopeia (USP) standards for vapor transmission^16,17^. For all drugs, analytical chemistry testing measured only the amount of API in each product at each time point; no evaluation of degradation products was performed, and degradation mass balance was not determined.

### Effect of spaceflight on drug content

Du et al.^14^ reported that mean API content in 25 out of 36 medications in the spaceflight group fell below USP standards for labeled API strength (i.e., “failed”) after 880 days of storage, compared to 17 out of 36 in the control group at the same time point. Across time points, the number of formulations that failed to meet specifications for API content increased with the duration of spaceflight exposure. These results are the basis for the conclusion by the study’s authors that “…a number of formulations tested had a lower potency or percent content of API after storage in space with a consistently higher number of formulations failing USP potency requirement after each storage period interval in space than on Earth”^14^. This conclusion, based on dichotomization of quantitative API content results (i.e., pass/fail outcomes), has been repeatedly cited as evidence that latent factors associated with spaceflight increase the risk of drug failure.

**Fig. 1:**
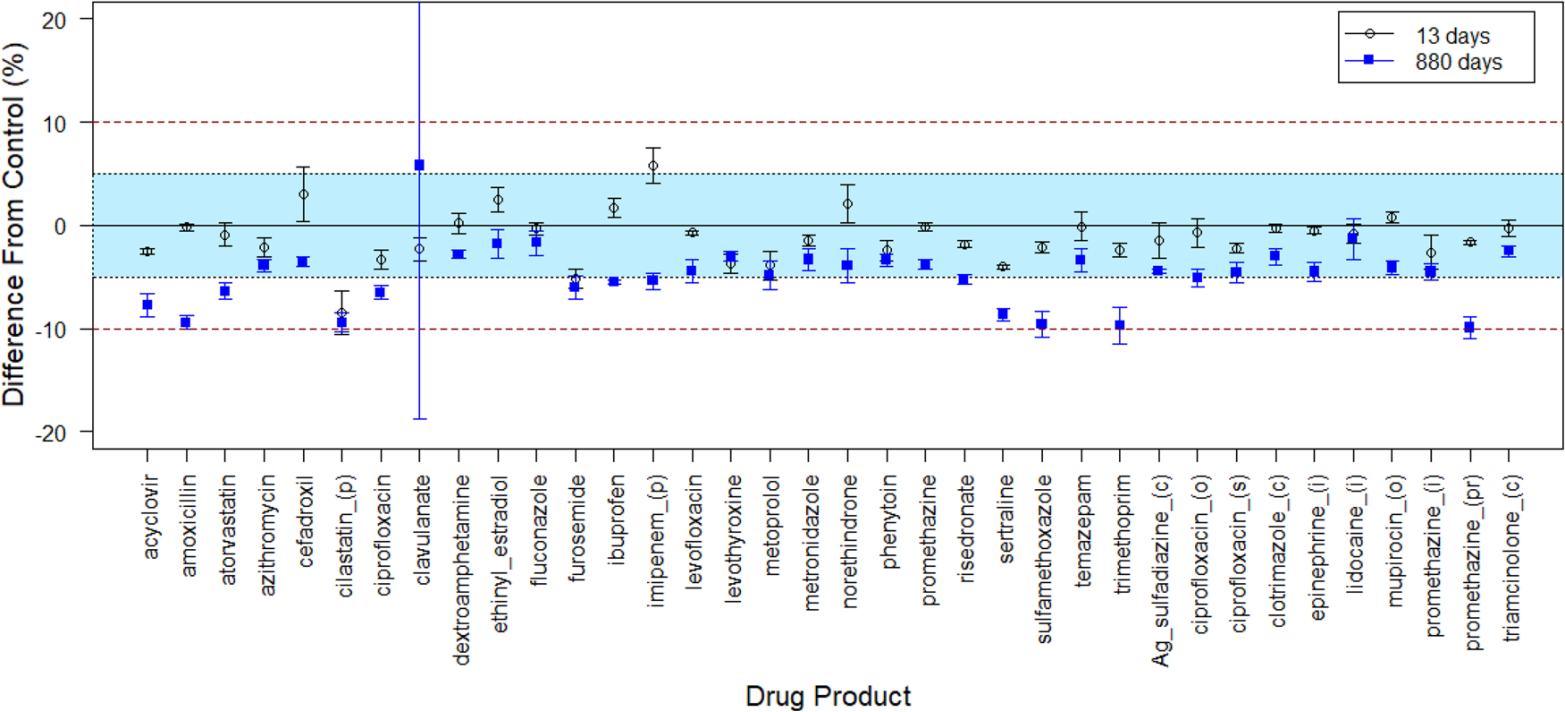
Relative change in API strength at 13 and 880 days of space flight. Plot of the difference between pair-matched control and spaceflight drugs stored for either 13-days (○) or 880-days () samples. Values are calculated as the difference of API potency (%) in control – API potency (%) in matching spaceflight samples. Error bars reflect ± one standard deviation (sd) to display estimated uncertainty. The blue shaded area represents a difference of ± 5% in API potency; the dashed line represents a ± 10% difference in potency. A value of zero indicates the API potency (%) of the spaceflight sample and its corresponding matched control are equivalent. (i) injectable, (c) cream, (o) ointment, (s) suspension, (pr) suppository, Ag silver.

Quantitative analysis of the drug API content reported by Du et al.^14^ provides substantially more insight into the effect of spaceflight on drug stability than the original qualitative evaluation. Across all drugs at the 13-day time point, the difference in API content between spaceflight samples is within ±5% of control content for most drugs (34 of 36) (Fig. 1). At 880 days of spaceflight storage, 39% of flight-exposed drugs (14 of 36) remain within ±5% of control potency, and no drug has a loss of API exceeding 10% of control amounts. (To be clear, this is control level ± a range of 10%, not a multiplicative percentage.) Taken together, this supports a conclusion that the effect of spaceflight exposure on drug stability is relatively small overall (Supplementary Table 2).

Intuitively, if spaceflight increases the rate of API degradation (i.e., accelerates chemical reaction rates of APIs), then it should be expected that the *difference* between terrestrial and spaceflight samples should be negligible at the very early 13-day time point compared to the difference expected after 880 days of storage^18^. However, at the 13-day time point a surprising 50% of all drugs in the flight group (18 of 36) have significant (p < 0.05) decreases in API content compared to matched controls (Supplementary Table 1). The mean decrease in API content for the flight samples compared to the terrestrial samples at the 13-day time point is 1.18 ± 2.5%, which equates to a rate of API loss of 0.09%/day. By comparison, the mean deviation from controls at the 880-day time point is 4.76 ± 3.01%, corresponding to a total mean change in potency from Day 13 to Day 880 of only 3.6% (4.76–1.18% = 3.6%), equating to mean rate of API loss of 0.004%/day for a storage duration of 880 days. Although the time interval between study days 14 to 880 is 60-times longer than the initial 13-day time point, the degradation ratio is only approximately 3-fold that observed over the period from Day 0 to Day 13, which equates to a relative degradation rate associated with spaceflight storage of 0.045, which is much less than unity, and suggests that a substantial portion of the reported degradation occurs prior to the earliest time point (Day 13), even though the duration of time after Day 13 accounts for 99.5% of the total period of spaceflight exposure. Hence, the loss of API reported for spaceflight samples is at least partially attributable to factors other than prolonged spaceflight exposure.

**Fig. 2:**
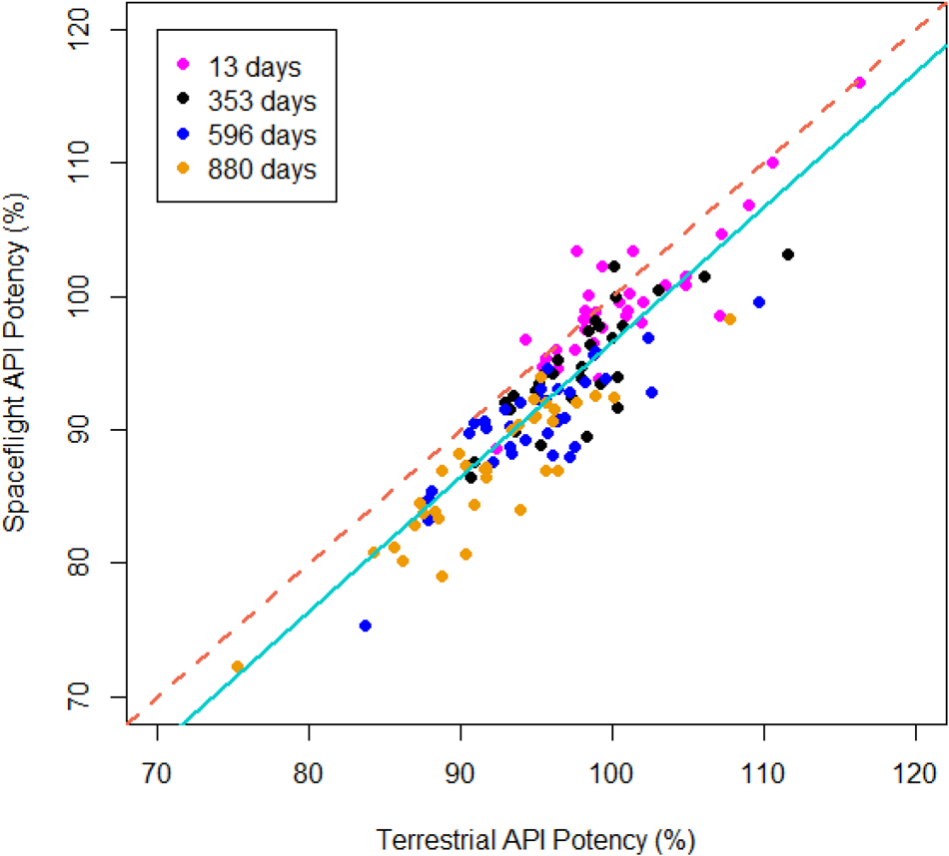
Potency relationship between spaceflight matched controls. Mean potency of spaceflight-exposed drugs are plotted versus matched controlled potency and are highly correlated (Pearson, r = 0.894). The slope of the solid regression line (cyan; slope = 1.012) is close to unity, (hashed; slope = 1 and intercept = 0).

Focusing on changes in API content occurring between study days 13 and 880 show that spaceflight and terrestrial drug potency are highly correlated (r = 0.894) with a nearly 1:1 correspondence (Fig. 2). Linear regression of these paired terrestrial and spaceflight potencies yields a slope coefficient of 1.012, which is virtually equivalent to unity and offset only by the y-intercept of −4.64%. On a per-drug basis, the Day 13 and 353 samples are slightly greater than unity, while the Day 596 and 880 samples are slightly less than unity. This indicates that the loss of API over the course of the experiment, aside from the location of the y-intercept, is very similar for control and spaceflight samples overall.

#### Effect of spaceflight on degradation rate

**Fig. 3:**
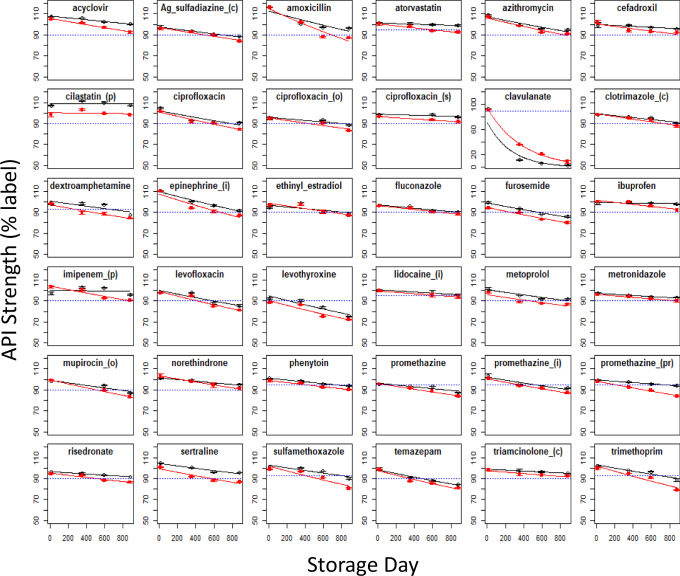
Plots of mean label API strength (± standard deviation) vs. storage time. Points are drug content (±sd) as a percent of labeled strength corresponding to 13, 353, 396, and 880-day time points for terrestrial controls (black) and spaceflight (red) APIs, as published by Du et al. (2011). Superimposed lines are first-order regression models for each mean drug concentration. The blue hashed line indicates the minimum USP standards. (i) injectable, (c) cream, (o) ointment, (s) suspension, (pr) suppository. Confidence intervals were not calculated, and statistical regression analysis was not performed because independence of replicate tests could not be confirmed based on the methods presented in Du et al.^14^.

Estimates of chemical degradation rates are crucial to enabling a mechanistic understanding of how API degradation is influenced by environmental conditions and to provide predictive insight for estimating drug strength over time. The FDA and the European Medicines Agency (EMA) accept that drug degradation rates are typically represented by linear kinetics; most commonly first-order reaction rates^18^. Regression models can test the null hypothesis of equality of slope or intercept relative to a control sample. However, the very low standard deviation for each drug (Supplementary Table 1) raises an uncertainty regarding the independence of replicates that justifies treating each mean value as a single independent observation. For each drug in this study, rates of degradation are visualized as a series of scatter plots upon which fitted first-order curves for control and flight samples are superimposed (Fig. 3). From these plots, it can be observed that for many of the APIs, the control and spaceflight degradation curves are close to parallel with the two curves primarily offset by variability in the location of the y-intercept (i.e., the API strength at time zero). These plots illustrate that, for most of the tested drugs, spaceflight contributes minimally to the degradation, as summarized numerically in Supplementary Table 3, which also provides extrapolated estimates of API half-life under control and flight conditions, as well as an estimation of API remaining at three years.

Inspection of the rate relationships shows that spaceflight is generally associated with a small increase in the rate of API loss. Rate ratios comparing terrestrial and matching spaceflight samples greater than 1.0 (Supplementary Table 3) indicate that the spaceflight rates of API loss exceed that of matching control samples. For 30 out of 36 drugs, rate ratios are less than 2-fold, ranging from 0.69 to 1.97. Only 2 of 36 APIs exhibit spaceflight degradation rates exceeding 3 times the terrestrial control rate: ibuprofen tablets (7.02-fold), and lyophilized imipenem for injection (9.43-fold). Cilastatin is unique in that it exhibits no degradation over time and a negative rate ratio that may be attributable to the combination of high stability and analytical variability. Conversely, clavulanate, which was by far the least stable drug tested, is the only drug where stability is greater under spaceflight conditions than under terrestrial conditions (rate ratio = 0.69). Elevated rate ratios are expected to be associated with drugs that are most susceptible to degradation during spaceflight. It is noted that most of the drugs with the highest rate ratios also have control degradation rates that are extremely slow, as indicated by the corresponding estimated half-life. For this reason, a small increase in degradation rate in the flight samples in terms of mass content, produces a large change in the rate ratio for some of these drugs.

Half-life is an intuitive metric for evaluating concentration-dependent loss of API over time. Calculated half-life estimates (Supplementary Table 3) show that most of the spaceflight APIs (24 of 36) have half-lives exceeding a decade, with the remaining 12 drugs having half-lives shorter than ten years (controls are 31 and 5, respectively). Extrapolated, the rate of API content loss suggests that, under repackaging and storage conditions analogous to those currently used by NASA operationally, the potency remaining for most drug products in both the control and spaceflight treatment groups at the end of a three-year exploration space mission falls below 90% of the label strength (Supplementary Table 3). Although it is certainly not ideal to rely on deteriorated pharmaceuticals to treat medical conditions during exploration space missions, most of the tested drugs would have adequate API content remaining to achieve therapeutic efficacy. However, it is preferable to understand the mechanistic factors contributing to degradation which enables countermeasures to be implemented that avert the clinical risk of degraded medications in the first place. It is noted that neither current nor any previous drug repackaging practices are protective for vapor or light transmission, as defined by USP^16^.

**Fig. 4:**
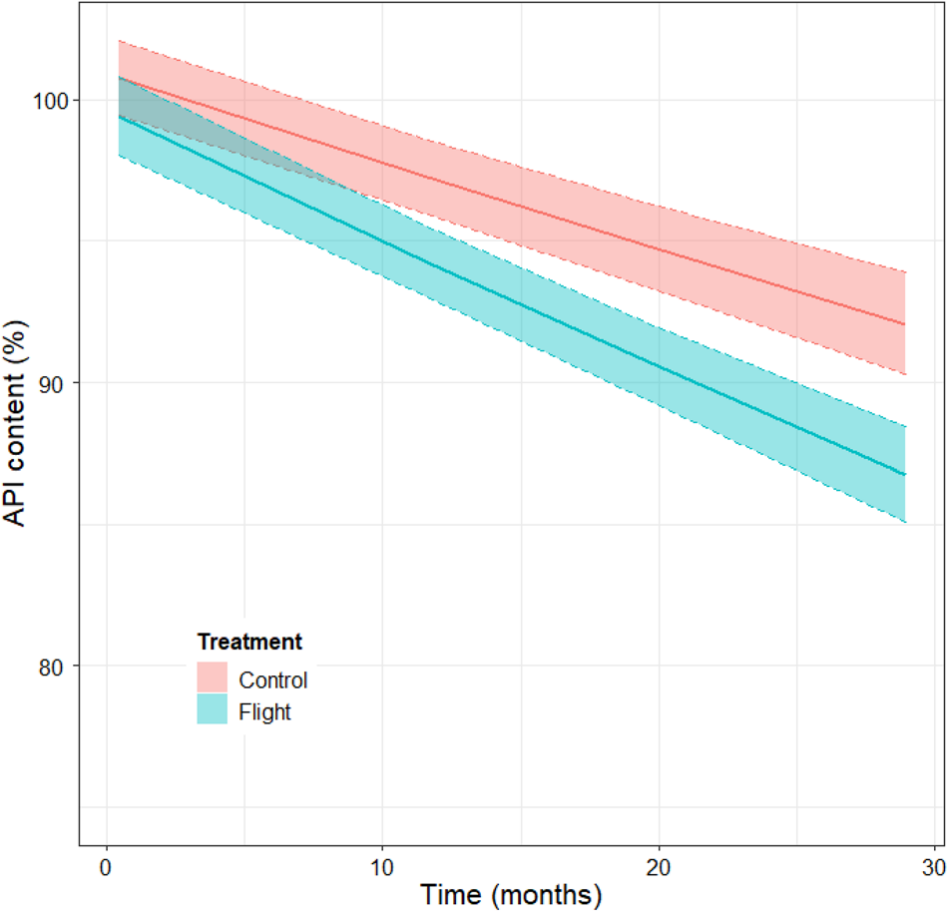
GEE model results for API potency. The shaded bands represent 95% confidence intervals of the regression mean response. Note the scale of the ordinate axis is truncated at 75% API content.

Each paired set of flight and control drugs (i.e., within-drug comparisons) is independent of all other paired sets of medications (i.e., between-drug comparisons) and can therefore be collectively analyzed to estimate an overall effect of spaceflight on drug stability. For example, the measured contents of a promethazine tablet at one-time point are not independent across other time points because the content at a later promethazine time point depends on content at earlier time points; however, the content of promethazine at any timepoint is entirely independent of levofloxacin and other tested drugs, which enables these independent temporal data to be collectively modeled. Visual inspection of the individual fitted regression plots (Fig. 3) collectively suggests both slope and intercept variability across APIs contributes to differences in API levels observed between control and spaceflight samples. The evaluation of terrestrial and spaceflight treatments is analogous to whether or not results from independently performed stability tests can be combined under FDA shelf life stability testing guidance^18,19^. Linear mixed-effect regression models have been used as one approach for such drug stability evaluationshypotheses^20,21^. However, a fundamental assumption of the mixed-effect regression models is that slopes and intercepts for each entity (i.e., drug product) are random and normally distributed. Since the drugs tested by Du et al. were arbitrarily selected for testing based, in part, on heuristic operational considerations, the normality of random slopes should not be assumed. Generalized estimating equation (GEE) models are an alternative approach that do not assume anything about random effects but do account for cluster correlation for each drug over time. The use of an exchangeable correlation structure allows for a single correlation parameter for all pairwise responses within an API. Thus, the model provides a population-level estimate of longitudinal drug potency accounting for clustered correlation. Here, we assume that different APIs, and potentially different drug formulations containing the same API (e.g., tablet, injectable), have different susceptibilities for degradation over time. Hence the postulated GEE model includes a variable for storage time (in units of months of storage), a factor for the treatment group (control or flight), and a clustering variable (API). In addition, an interaction term is also included in the model to account for the combined effects of storage time with the treatment group (flight vs. control). This interaction is mechanistically justified since storage time cumulatively increases the exposure of an unprotected API to environmental factors (e.g., humidity, CO_2_, ionizing radiation), or combinations of factors that individually or synergistically contribute to API degradation. This contribution was evaluated using interaction plots and model selection prior to including this effect as a GEE model parameter (Supplementary Fig. 2). The GEE model results show that time, and the interaction of time and storage conditions, are the most significant coefficients in the model (*p* < 2e-16), with the effect of spaceflight itself being a less significant contributor to degradation (p < 0.0014). The collective first-order degradation rate for 35 APIs (excluding clavulanate) under terrestrial conditions is −0.00317/month (t_½_ = 219 months). These findings compare to a degradation rate of −0.00478/month (t_½_ = 145 months) for spaceflight samples, which equated to a ~1.51-fold (51%) increased rate, or an *additional* rate of −0.0016/month over the baseline rate. Figure 4 compares the overall first-order degradation of all drugs stored terrestrially to similarly maintained control samples with pertinent GEE model coefficients provided in Table 1, and the marginal supporting effects are provided in Supplementary Table 4. Converted to an arithmetic scale, this equates to an additional ~0.2% loss of API content per month relative to the terrestrial baseline when averaged over the total duration of the experiment. The cluster correlation is estimated to be 0.651 ± 0.0703, indicating substantive temporal concordance within a cluster (i.e., APIs), which strongly supports the GEE approach for modeling cluster correlation. Not surprisingly, the same analysis performed using mixed-effect regression yields very similar results (Supplementary Note 1).

**Table 1:** GEE model regression coefficients^a^.

Term	Coefficient	Standard error	Wald statistic	*p*-value
Intercept	4.61E + 00	6.66E-03	481502.6	<2e-16
Storage time	−3.17E-03	3.11E-04	103.9	2.00E-16
Treatment (flight)	−1.27E-02	4.98E-03	10.2	0.0014
Interaction (time *treatment)	−1.61E-03	1.79E-04	80.8	2.00E-16

^a^Model coefficients represent Ln(response).

#### Time-dependent failure rate

One measure of drug “failure” occurs when the API content of a drug product does not meet the minimum percentage of labeled strength, which, in the United States, is established by USP drug specifications. The overall risk of drug failure is a NASA concern for long-duration spaceflight, especially for deep space exploration missions where resupply may be difficult or impossible. USP specifications of drug API content are minimum API content thresholds that serve as dichotomous pass/fail classifiers. USP limits are based primarily on reasonably achievable manufacturing quality and analytical performance; they are not quantitative metrics of pharmacodynamic potency, therapeutic efficacy, or toxicological risk. For stability testing of pharmaceuticals, the lower 95% confidence interval of a regression model is used to describe degradation rate as a function of time and is used to predict the retest period and shelf life^18,22,23^. In this paper, the intersection of the lower 95% confidence interval of measured API potency with the lower limit of the USP quality range is used as the threshold to classify each drug product as “pass” or “fail”. This assumption is more conservative than using mean values (as was done by Du et al.^14^) because it causes the estimate of drug content to intersect with USP thresholds at an earlier time than does the mean potency. Again, a limitation of this analysis is that variance in the API measurement (Supplementary Table 1) is likely only analytical variability and not a full representation of experimental variability.

**Fig. 5:**
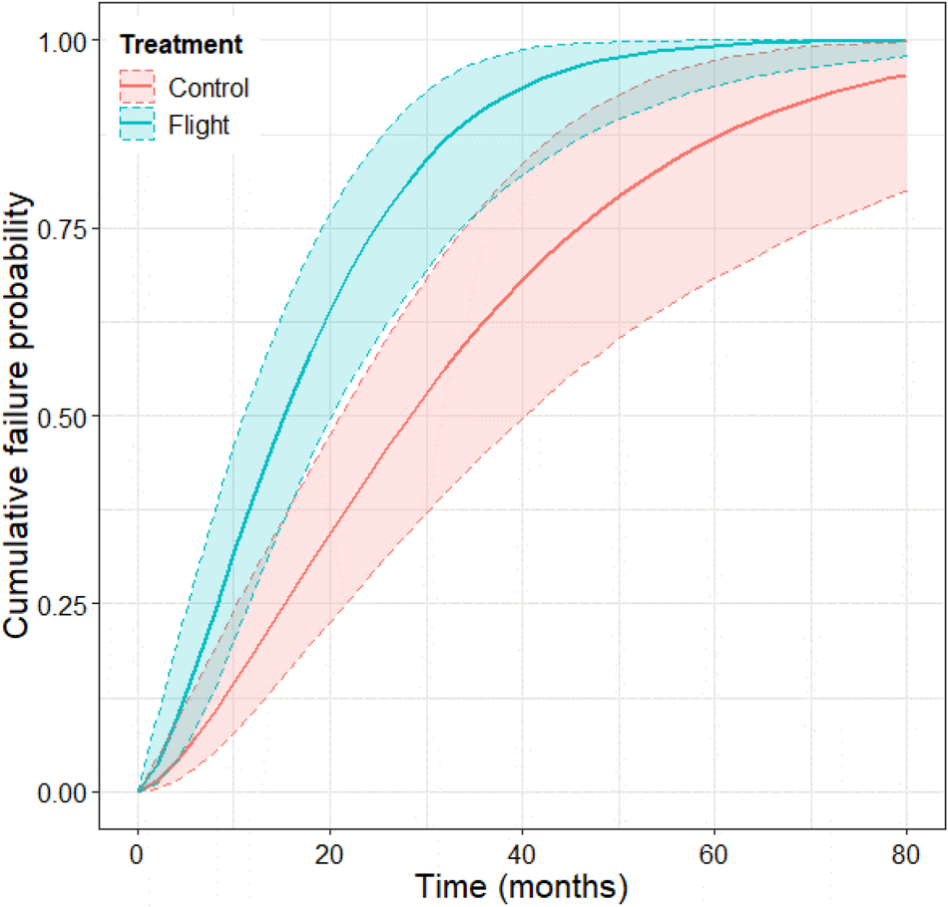
Accelerate failure time model results. The shaded bands represents 95% confidence intervals of the regression mean response.

Failure time analysis focuses on *when* a failure event occurs rather than *if* an event has occurred, as in survival analysis. The risk of drug failure is the probability that a drug will fail to meet or exceed compendial specifications for API content at any point, and this probability increases over time. Figure 5 illustrates the cumulative posterior median failure distribution of terrestrial and spaceflight drug samples uncorrected for the mean difference between control and spaceflight API strength discussed earlier. Both spaceflight and terrestrial conditions exhibit a rapid increase in failure probability during the earliest months of storage in this experiment. The risk of failure with spaceflight storage is superimposed upon, but lower in absolute value, than the baseline risk of failure observed for the terrestrial controls.

Overall, the time to failure for drugs exposed to spaceflight, based on assayed API potency, is approximately half (0.55) that of a drug under terrestrial conditions (95 % CI = [0.37, 0.82], p = 0.0038) if a proportional hazard is assumed. From the Bayesian model, the median estimated time to failure is 28.6 months (95% CI, 21.0–40.9 months) for terrestrial storage and 15.3 (95% CI, 11.2–20.1 months) for spaceflight. Based on the posterior survival distributions, probabilistic failure estimates for specific storage times are provided in Table 2. Whether the probability of failure for the baseline terrestrial samples is increased due to environmental exposure as a result of repackaging or inherent chemical instability cannot be determined directly from this study since matching controls in unopened manufacturer packaging were not tested.

**Table 2:** Probabilities of Drug failure through specific time durations.

Median	Storage duration (Months)			
Probability ± SD	1	12	24	36
Terrestrial storage	0.007 ± 0.005	0.188 ± 0.049	0.426 ± 0.072	0.627 ± 0.085
Spaceflight storage	0.0017 ± 0.001	0.388 ± 0.066	0.729 ± 0.065	0.898 ± 0.049

#### Concordance with anecdotal spaceflight stability studies

The study by Du et al.^14^ is the only investigation to date that includes multiple drugs that were not repackaged (14/36 drugs). Of these drugs, ten are an assortment of nonsolid formulations, including solutions, ointments, creams, and a suppository. The remaining four APIs that were not repackaged were solid combination products: imipenem with cilastatin (lyophilized powder for injection) and ethinyl estradiol with norethindrone (blister pack oral tablets). After 880 days of spaceflight, only 2 of 14 drugs (~14%) remaining in manufacturer packaging deviate from their corresponding controls by more than 5%, compared to 9 of 22 (41%) repackaged medications. Similarly, for those drugs that failed based on API content during the 880-day storage period, the mean failure time for drugs that were not repackaged is 707 days (*n* = 8), of which most are non-solid formulations (the one exception is ethinyl estradiol). This compares to an average failure time of 633 days for repackaged drugs (*n* = 12), all of which are either capsules or tablets. Solid formulations typically have longer shelf lives than non-solid formulations of the same drugs. Since it is well established that repackaging can adversely affect the stability of drug products^5,24–26^ the more frequent and earlier failure of solid formulations suggest that repackaging could be an important contributor to drug degradation reported in spaceflight studies.

**Fig. 6:**
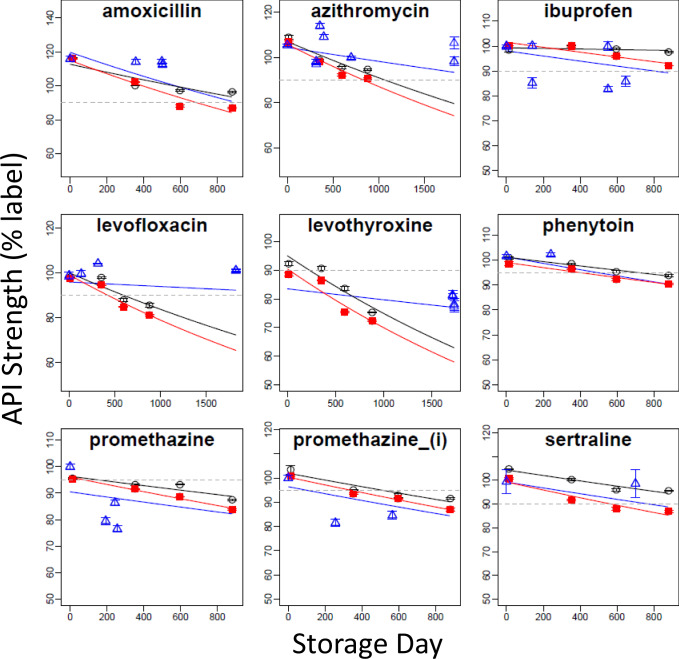
Least squares degradation trends for 8 APIs (9 formulations). The blue solid line is the overall trend for all available spaceflight data, including results from Du et al.^14^ and sui generis results from opportunistic studies. Triangles are mean ± sd. of API potency from opportunistic studies. Data points correspond to mean ± sd. of API potency in control (black) and spaceflight (red) samples from Du et al., with superimposed least squares trend lines. (i) injectable; other medications are oral formulations.

In addition to the study by Du et al., five smaller descriptive opportunistic studies of spaceflight drug stability have been performed (Table 3)^9–11,13^. Among these studies, none include initial baseline API measurements prior to long-term spaceflight exposure or terrestrial lot-matched controls. Of these studies, only the study by Wotring^12^ is published, whereas the other four studies are NASA reports (extracted data from these studies are provided as described in the Data Availability section). The range of APIs tested among these five studies is much more limited than that of Du et al; however, there is a much greater focus on characterizing impurities, albeit without lot-matched terrestrial controls. Three of these studies include matched manufacturer controls for each spaceflight exposed medication, however, these controls are from different lots with different expiration dates^9–11^, and one study includes both unmatched and *some* lot-matched controls^13^. Across the six studies (inclusive of Du et al.), a total of nine medications (Table 3, bolded) intersect with the list of medications tested by Du et al. Of these, ibuprofen is the most commonly tested drug, having been evaluated in four out of six spaceflight studies. Two medications are shared across the five studies in Table 3 that are not included in the Du et al. study (‡ superscript), with the remaining drugs having been evaluated in only a single study. The study by Khan and Wotring^13^ is distinct from the other four studies listed in Table 3 in that the three medications tested are identical medications originally tested by Du et al. several years earlier. In this respect, these results are independent measurements on the same Du et al. samples but following a considerably longer period of post-flight terrestrial storage. Figure 6 summarizes data from all studies as scatter plots of mean API levels (±SD) for the nine drug products (eight APIs). A trend line incorporating all available data for each medication (blue line) is plotted to illustrate the overall pattern for loss of API content with slopes provided in Supplementary Table 5. For reference, the trend lines for the matching control and spaceflight medications from Du et al. are also provided as described in Fig. 3. A key observation from these composite plots is the large variability in measured API content across studies.

**Table 3:** Opportunistic Spaceflight Medical Stability Studies.

Study	Cory et al.^11^	Cory et al.^10^	Wotring^12^	Wu and Chow^9^	Khan and Wotring^13^
**Space Platform**	ISS	ISS Medical kit	ISS Medical kit	ISS Medical kit	ISS (retest of Du samples)
**Publication status**	NASA Report	NASA Report	Published	NASA Report	NASA Report
**No. of drug products/APIs**	3/3	5/5	9/9	4/3	3/3
Independent replicates	*N* = 10	*N* = 9 (Phenytoin)	*N* = 4 or 5	*N* = 3	*N* = 3
		N = 10 all others			
**Control time points**	One per API; no storage time points	One per API; no storage timepoints	No controls	One per API; no storage timepoints	Mixed. Some Correspond to spaceflight time points, some do not
**Matched or unmatched design**	Unmatched	Unmatched	NA	Unmatched	Each flight sample has lot-matched control
**Independent replicates**	Independent between lot analyses for the same drug; unclear if within lots replicates are independent (n = 10 replicates)	Independent between lot analyses for the same drugs; unclear if within lot are independent (n = 9 or 10 replicates)	Independent replicates of a single lots. (n = 4 or 5 replicates)	Independent replicates; likely tested a single lot for each drug and timepoint (n = 3)	Independent replicates, analytical analysis performed at FDA per USP monograph and repeated on separate days.
**No. of lots tested**	Three separate lots/flight medication + an unmatched control for each medication	One lot/flight medication + unmatched controls for each medication.	Not discussed; likely one lot per medication	Different lots for each time point/control; no indication samples were from the same manufacture at each time point	Different lots tested for each drug or time point (one control is pair-matched to one flight sample)
**Repackaged**	All flight samples repackaged; control’s packaging not described.	All flight samples repackaged; control’s packaging not described.	All medications were repackaged into zip-lock bags.	All flight samples repackaged; control’s packaging not described.	All flight samples repackaged. Samples evaluated here are identical to Du et al. 2011
**APIs Tested**	**Amoxicillin**^**b**^, Aspirin^a^, Pseudoephedrine^a^	**Levofloxacin**, **Ibuprofen**, **Phenytoin**, Valacyclovir, **Sertraline**	Aspirin^a^, Acetaminophen, **Ibuprofen**, Loratadine, Loperamide, Melatonin, Modafinil, Pseudoephedrine^a^, Zolpidem	**Promethazine**^**c**^, **Azithromycin**, **Ibuprofen**	**Levothyroxin, Levofloxacin, Azithromycin**
**Analytical instrumentation**	HPLC with electrospray LC-MS	HPLC with UV or electrospray LC-MS	HT HPLC with PAD detector or UV DAD detector	UPLC‐MS/MS	HT HPLC with DAD
**Analytical performance (API)**	Not discussed	Not discussed	Not discussed	Accuracy, precision, LLOQ, LLOD	Accuracy, precision, specificity, LLOQ, LLOD

^a^APIs shared across some oppertunistic studies that were not amongst drugs tested by Du et al.^14^.

^b^Du et al. tested amoxicillin in combination with clavulanate, whereas Cory et al.^11^ tested amoxicillin as a single API drug product.

^c^Both tablet and injectable formulations.

**Bolded** APIs are amongst the drug substances tested by Du et al.^14^, although product manufacturers may be different between studies.

*API* active pharmaceutical ingredient, *ISS* international space station, *HPLC* High-performance liquid chromatography, *UPLC* Ultra-performance liquid chromatography, *MS/MS* tandem mass-spectroscopy, *DAD* Diode Array Detector, *PAD* Pulsed Amperometric Detector, *LLOQ* lower limit of quantitation, *LLOD* Lower limit of detection, *LC-MS* liquid chromatography-mass-spectroscopy.

Across all studies, five out of the nine intersecting drugs exhibit higher amounts of API in the follow-up studies than were reported by Du et al. at similar or earlier time points. The opportunistic studies of ibuprofen yield API percentages that bracket those reported by Du et al., with lower levels of API at all time points reported by Wu and Chow^9^ and higher levels reported by both Wotring^12^ and Cory et al.^10^. Both the oral and injectable dosage forms of promethazine were reported by Wu and Chow^9^ to have lower amounts of API than was reported by Du et al. Among the nine composite models, spaceflight degradation rates are reduced in five models (i.e., the rate of degradation is slower) when all data were considered; only phenytoin exhibits an apparent increase in the estimated rate of degradation. Rate estimates for amoxicillin, ibuprofen, and injectable promethazine (the latter being the only drug maintained its original manufacturer packaging) are relatively unchanged despite large variations in measured API content.

## Discussion

Several studies and review articles have suggested, based primarily on anecdotal analyses, that long-term exposure to spaceflight may facilitate drug degradation and increase the risk of therapeutic failure^14,27–29^. We have reviewed these studies and performed a quantitative analysis of available data to characterize the overall effect of spaceflight on the rate of API loss and the risk of drug failure and have attempted to benchmark results across studies. Our analysis suggests that degradation observed in terrestrial control samples is the dominant factor contributing to overall loss of API content, and that spaceflight storage contributes an additional but much smaller effect.

It is common practice for NASA to remove solid oral drug products form their manufacturers’ containers, and repackaging them into light weight packaging to reduce mass and volume. Existing studies have not directly investigated how the spaceflight environment influences API degradation or which attributes of some drug products increase susceptibility to degradation. Not unexpectedly, most of the drugs tested across all the NASA-supported stability studies are solid oral medications repackaged into nonprotective packaging. To date, no NASA study has tested the effect of non-protective repackaging on drugs stability or evaluated how nonprotective packaging contributes to spaceflight drug degradation relative to the same medications in their sealed manufacturer container.

The effect of repackaging is evident from the high failure rate of drugs in the *terrestrial control* group reported by Du et al (2011). Of the 34 drugs tested, 11 failed, based on API content, *prior* to the label expiration date. Of the 11 failed medication, 9 were repackaged orals drugs while the remaining two were non-solid topical medications (suppository and cream). The fact that 41% of *control* solid oral drug products prematurely failed (i.e., failed to meet UPS drug content prior to their labeled shelf life) is incredibly consequential because manufacturers guarantee product to meet quality specifications throughout their entire shelf life. Since the terrestrial group was exposed only to controlled environmental conditions that were consistent with label storage requirements, the high failure rate of control samples suggests repackaging is the most likely factor contributing to the loss of potency. Currently, the effect of drug repackaging on drug stability is not a concern for LEO missions because ISS is readily resupplied; however, resupply will not be possible for exploration space missions. Therefore, the effect of drug repackaging on spaceflight degradation must carefully evaluated to assure acceptable levels of drug stability throughout the full duration of an exploration mission.

At the first time point (13-days) some spaceflight drugs exhibit a pronounced decrease in potency with up to 8.5% less API compared to matched control (Fig. 1, Supplementary Table 2) and a mean difference between the two storage conditions of 1.35% (Supplementary Table 4). If the mean difference in potency observed at the 13-day time point is interpreted as the rate of change (i.e., 1.35%/13 days = 0.104 %/day), and if this rate were maintained throughout the experiment, then on average the spaceflight drug products would contain substantially less than half of the labeled amount of API at the conclusion of the 880-day experiment. Since this is not the case, it can be concluded that the rate of API loss prior to the first timepoint is much greater than the rate of loss from Days 13 to 880 (Supplementary Table 2). The loss of API prior to Day 13 must be attributable to either factors not directly associated with spaceflight storage (e.g., repackaging or handling, sample processing), or exposure of the samples to extreme environmental conditions, in particular elevated temperature, which accelerates chemical reactions. This difference between control and spaceflight samples persists throughout the experiment and appears to affect most medications, similarly, as exemplified by cilastatin (Fig. 3). After Day 13 the API content for all drugs is linear with time and is well characterized by pseudo first-order reaction rate.

On average, the amount of API remaining after spaceflight storage was statistically less than corresponding lot-matched controls across all the medications tested. However, it is important to distinguish a statistically significant change from a clinically significant one. Statistical significance assumes sample replicates are independent measures and, if this assumption is violated (e.g., pseudo-replication), the statistical tests are biased by artificially low sample variance, which increases the likelihood of falsely rejecting a true null hypothesis, a type 1 error (false-positive result). In such a case, it would be erroneously concluded that treatment samples are “significantly” different from controls when, in fact, they are not. Although API content of most spaceflight medications is significantly less than corresponding controls, especially at the later time points, the magnitude of the difference is relatively small. On average, the difference between terrestrial and spaceflight samples increases with storage time to 2.29 ± 5.55% after 353 days of storage, 3.93 ± 4.09% at Day 596, and 4.76 ± 3.01% at Day 880. Consequently, most spaceflight samples fall within 5% of their respective terrestrial control at 880 days (2.4 years) of storage, and all medications in the spaceflight group were within ±10% of the matching controls (Fig. 1). Thus, while most spaceflight-exposed drugs have a significant loss of API at the 880-day time point and fail to meet the USP standards for API strength, corresponding controls samples also undergo a similar loss of API content. For this reason, the clinical efficacy of spaceflight medications would likely not differ from similarly aged terrestrial controls.

It was interesting to integrate the results of Du et al. with those of other spaceflight stability studies where equivalent drug products were also tested. Across these studies, only nine drug products intersect with the list of drugs tested by Du et al. (bolded text in Table 3). There are several study design challenges that hinder the comparison of results across spaceflight stability studies, including an absence of baseline results on study Day zero, cross-sectional (single point) rather than longitudinal study design, and an absence of terrestrial lot-matched reference controls. Although, for some drugs, API potency and impurity content after prolonged spaceflight differ from both unmatched terrestrial reference samples (different manufacturing lots and expiration dates) and from labeled strength, the absence of a lot-paired sample design means that no determination can be made about the effect of spaceflight exposure relative to quality changes during the same period of storage under controlled terrestrial conditions. In absolute terms, however, we can use these anecdotal studies as independent benchmarks to contextualize API content for spaceflight-exposed drug products similar to those reported by Du et al.

When individual least-square trend lines for the drugs tested by Du et al.^14^ are updated with potency data of equivalent drugs tested in other studies, spaceflight is not a significant (*p* ≤ 0.05) predictor of degradation for any drug. These findings are not surprising given the large variability in potency for the nine APIs (Fig. 6; Supplementary Table 5). There are at least a couple of potential explanations for the apparent variability in API potency across different studies. One possibility is variable extraction efficiency across equivalent drug products from different manufacturers. Different studies have used different brands of equivalent drugs. For example, Cory et al.^11^ tested Levofloxacin from Sandoz whereas Khan and Wotring^13^ tested an equivalent Janssen product (Supplementary Table 5). USP methods are cited by all spaceflight stability studies as the procedures used for sample preparation and analysis of API content. However, analytical methods are submitted to the USP by a product’s innovator or sponsor, and procedures are optimized for that sponsor’s specific drug product. Inactive ingredients used to formulate equivalent drug products can vary widely among manufacturers, and different excipient ingredients have the potential to interfere with compendial procedures for sample processing, and may affect the recovery of the drug substance from the matrix^30,31^. Reliance on compendial methods without adequately demonstrating method suitability for a particular drug product, as a result of either insufficient validation or verification, results in less reliable estimates of drug content. A second possibility for the variability in potency across spaceflight drug stability studies is that drug repackaging practices may be different across studies; hence, environmental exposure at different points in time may vary with differences in repackaging methods. Most of the studies listed in Table 3 do not describe how drugs were repackaged but it is assumed zip-lock baggies were used, aside from Khan and Wotring^13^ and Du et al.^14^.

USP specifies standards for protective drug packaging that are intended to assure that the container in which a drug product is packaged is suitable for maintaining potency through the drug’s expiration date^16,17,32^. The key function of suitable packaging are protecting the drug product from moisture or UV light. Du et al. described repackaged medication containers as cylindrical “polypropylene” containers without description of the container closures. The operational procedure for current and upcoming exploration missions is to repackage medications in re-closable zip-lock bags. Zip-lock bags are not consistent with USP guidance for vapor transmission or UV radiation protection^16,33^ (personal communication with the manufacturers). Both zip-lock bags and polypropylene containers without sealed closures allow the contents to equilibrate with the ambient atmosphere within days, even if the packaging remains unopened. Since current packaging procedures for solid drug formulations are not protective, degradation rates calculated for both terrestrial and spaceflight samples reflect exposure to atmospheric factors (e.g., humidity, CO_2_, oxygen) capable of promoting degradation of susceptible drugs. The difference in degradation rates for spaceflight and terrestrial samples is likely attributable, at least in part, to differences in atmospheric factors between the two storage environments. Latent factor(s) contributing to increased failure risk of spaceflight samples remain the subject of speculation, and elucidation of the relative contribution of these factors to the risk of drug failure should be an area of future investigation for NASA. Latent factors may include atmospheric contaminants such as solvents, reactive substances, CO_2_, ammonia, as well as logistical considerations such as storage location and temperature aboard the spacecraft, pre- and postflight storage and sample transport conditions.

Of the thirty-six APIs tested by Du et al., only potassium clavulanate was significantly *more* stable (p < 0.05, two tailed t-test) during spaceflight compared to matched controls at all time points after 13-days. Clavulanate also exhibited, by far, the most significant degree of degradation among all the drugs evaluated under either storage condition. At the 596-day time point, clavulanate was 6.6 ± 0.16% and 21.1 ± 1.1% (*p* = 0.002) of label strength for the control and spaceflight samples, respectively. At the 880-day time point clavulanate potency was only 3.3 ± 0.31% and 9.1 ± 2.07% (*p* = 0.04) of label strength for control and spaceflight, respectively. Hence, clavulanate is an extreme outlier in terms of the magnitude of API lost (Supplementary Fig. 1). Chemically, clavulanate is hygroscopic and highly susceptible to pH-dependent hydrolysis^34–38^. Relative humidity (RH) is a well-established facilitator of drug degradation and mediates the degradation of potassium clavulanate in solid formulations^37,39,40^. Du et al. reported that, on average, terrestrial RH levels were somewhat greater than spaceflight levels for matched samples. Since the tablets containing clavulanate were repackaged into containers that did not assure protection from atmospheric factors, the higher terrestrial RH may have facilitated hydrolysis of terrestrial samples. In addition, the partial pressure of atmospheric CO_2_ aboard ISS is in the range of 2.3–5.3 mmHg, which is approximately 10-fold greater than the terrestrially CO_2_ level of approximately 0.3 mmHg at sea level^41,42^. Hydrolysis of clavulanate is inhibited at acidic pH^34,35^. Hygroscopic pharmaceutical ingredients, such as clavulanate, adsorb atmospheric moisture resulting in slow dissolution of the API by water within the microenvironment of the drug^43–45^. In the presence of water, CO_2_ forms carbonic acid, at an equilibrium according to Henry’s law^46^. It is conceivable that the slower rate of clavulanate degradation observed during spaceflight is, in part, related to carbonic acid (H_2_CO_3_) acidification in the microenvironment where clavulanate hydrolysis occurs. Thus, the combined effect of lower RH and elevated atmospheric CO_2_ during spaceflight may contribute to a rate of hydrolysis that is somewhat slower than that observed in terrestrial samples. Terrestrial atmospheric CO_2_ has been previously demonstrated to affect the stability of the drug sevelamer HCl^47^. The actual effect of CO_2_ on the stability of drugs sensitive to pH-dependent hydrolysis may be worth further investigation.

One limitation of the current analysis is that all NASA-sponsored drug stability studies have tested drug products from a single manufacturer/labeler and in many instances have not report the manufacturer of the tested product. It is well established that different drug brands can have significant variations in degradation rate and shelf life due to differences in formulation and packaging^8,13,48,49^. Testing only a single manufacturer’s product makes it impossible to characterize variability in API stability across equivalent formulations of a drug product. This information will be important where ingredients are used that may increase (i.e., antioxidants) or decrease stability of the drug substance (i.e., alcohols, esters) for exploration space missions^48,50^.

In summary, exposure of drugs to long-term LEO spaceflight appears to accelerate the degradation rate of some medications and increases the probability of drug failure based on API content. However, the additional risk of drug failure contributed by spaceflight is a fraction of the baseline risk observed for terrestrial controls. Although the factors contributing to increased degradation have not been established, it is noted that all spaceflight drug stability studies have focused primarily on drugs that were removed from the manufacturer’s containers and repackaged into containers do not protect medications from the ingress of atmospheric factors. Atmospheric factors (e.g., O_2_, CO_2_, RH) are well-established mediators of drug degradation. Since baseline degradation of terrestrial control samples accounts for the majority of API loss, repackaging is a simple and well-established explanation for time-dependent drug failure. Hence, differences in atmospheric composition between terrestrial and spaceflight storage, inclusive of pre-launch and post-flight logistics, likely contribute to the observed time-dependent differences in API content. However, the interaction of repackaging with storage condition has not been investigated during spaceflight. Future NASA studies of drug stability should focus on elucidating the role of the drug repackaging processes on drug stability and evaluate the benefit of protective packaging for susceptible drug products. For those APIs that are likely to be sensitive to atmospheric factors or ionizing radiation (in the range of exposure achievable during a long-duration exploration mission) an effort should be made to identify manufacturer brands or excipients that maximize storage shelf life to assure medication effectiveness for the duration of exploration space missions.

## Methods

A literature search was performed to identify all English language spaceflight drug stability studies (summarized in the accompanying Supplemental Methods) and ensure a complete dataset and inclusion of data. API content was quantified across the majority of spaceflight drug stability studies. Additional measures of drug stability, including impurities and physical characteristics, were only available in some studies and only for a few drugs. For this reason, this paper focuses on API content as the measure of drug stability.

The kinetics of API loss was evaluated based on both zero-order and first-order reaction kinetics. The selection process for the most parsimonious model is captured in Supplemental Methods.

### Statistical analysis

All statistical analyses were performed using open-source R statistical software (version 4.0.5) using R Studio (Boston, MA version 1.4.1106) as described in the Supplemental Methods section.

Generalized estimating equation (GEE) models and failure time analysis assume that each drug formulation is a distinct entity that requires independent processing (e.g., API extraction procedures) and analytical procedures (e.g., HPLC columns, buffer conditions, detector response factors). Therefore, each drug product represents an independent test. However, repeated measurements of API potency over the time course are not independent since these represent measurements of a specific drug lot over time and are expected to be correlated. GEE models account for temporal correlation for each individual unit (i.e., drug) and, as a result, are expected to give a better estimate of the population mean response across all tested drugs.

To evaluate the overall effect of spaceflight on drug stability and account for within-in-cluster correlation to temporal data, GEE models were used to estimate API content loss rate over time. The API content at each of the four timepoints for all 35 assayed drug products were used as input to the GEE model. GEE analysis was performed using the “geepac” package (version 1.3–2) for R software using the geeglm() function with “exchangeable” correlation structure and family = gaussian. APIs served as the clustering id. Analysis was supported by ggeffects (v. 1.1.1), emmeans (v. 1.7.2) MuMIn (1.43.17), ggplot2 (3.3.3), and lattice (v. 0.20–41) packages. Mixed-model regression was also performed, yielding similar results, as presented in Supplementary Methods.

Failure time analysis was performed to estimate the probability of drug failure as a function of time. Failure analysis is the inverse of a survival model that assumes a sequence of stresses, or just time itself, that incrementally increases the probability of failure. The drug content data exhibit left, right, and interval censored events. The sampling interval in the Du et al. (2011) study is approximately 9- to 10-months, which is not trivial given that the shelf life of many drugs is in the range of 1 to 3 years, which provides strong justification for accounting for censoring in the analysis^51^. In all instances, censored events are considered uninformative since the sampling scheme is based on operational flight schedules distributed at a roughly regular interval without any relationship to drug expiration or stability profiles. Additionally, for determining when drug failure occurs, we use the intersection of the lower 95% confidence interval limit (calculated from summary statistics) with the USP compendial threshold for API content, which is compliant with US Food and Drug Administration (FDA) guidelines for drug shelf life testing. Using the available mean and standard deviation for each time point, the lower 95% confidence interval limit on the mean was calculated as:

1 $$\bar x - t \ast \frac{\sigma }{{\sqrt n }}\quad (df = n - 1)$$

where $\bar x$ = the sample mean test values at time point *i*, t = the t-value of 95% CI (t = 4.303), σ = sample standard deviation and *n* = the number of observations (*n* = 3). Our analysis uses the current minimum USP threshold for API label strength for all drugs evaluated, not the USP (or author-assumed) thresholds used when studies were published or submitted to NASA.

It is noteworthy that for the GEE, mixed-effect model, and AFT model analyses, the drug clavulanate was excluded from the analysis. Clavulanate exhibited large variance among replicates and is a significant outlier in both the flight samples and terrestrial controls with degradation (Supplementary Fig. 1). This variance is far greater for clavulanate than any other drug (Fig. 1). Importantly, clavulanate is the *only* drug where the spaceflight sample appears to be *less* degraded than the terrestrial control. Therefore, removing this outlier is expected to increase the apparent effect of spaceflight (i.e., greater likelihood of rejecting the null hypothesis) in the overall model. This observation may represent a conservative and health-protective bias since it slightly increases the overall estimate of spaceflight drug degradation.

In addition to the methods described here, methods used in this manuscript are provided in the accompanying Supplementary Methods section.

## Supplementary information

SUPPLEMENTAL MATERIAL

## Data Availability

Data supporting this research are publicly available. Original data published by Du et al.^14^ are available as summary data from the publisher’s website (see Supplementary Table 3A, B of the Du et al. paper) and from Figshare 10.6084/m9.figshare.19394249. Unpublished opportunistic data are available from Figshare via the 10.6084/m9.figshare.19394252). All figures in this communication were generated using these data.

## Code availability

Analysis code in R software code can be provided upon request.
