# Supplementary material for: The effect of long-term spaceflight on drug potency and the risk of medication failure
Source: NPJ Microgravity. 2023 May 5;9:35. doi: 10.1038/s41526-023-00271-6 (PMC10163248; doi:10.1038/s41526-023-00271-6)
Supplement: Supplementary file 1 — SUPPLEMENTAL MATERIAL [file 41526_2023_271_MOESM1_ESM.pdf]

SUPPLEMENTARY INFORMATION
For
THE EFFECT OF LONG-TERM SPACEFLIGHT ON DRUG POTENCY AND THE RISK OF MEDICATION FAILURE

J.F. Reichard[1, 2, *], S.E. Phelps[3,4,5] , K.R. Lehnhardt[1,6], M. Young[1], B.D. Easter[1,7]

[1] NASA Johnson Space Center, Houston, TX

[2] Department of Environmental and Public Health Sciences, University of Cincinnati, Cincinnati, OH

[3] KBR, Houston, TX

[4] Department of Public Health & Preventive Medicine, University of Texas Medical Branch, Galveston, TX

[5] Departments of Emergency Medicine & Neuroscience, Emory University, Atlanta, GA

[6] Department of Emergency Medicine and Center for Space Medicine, Baylor College of Medicine, Houston, TX

[7] Department of Emergency Medicine, University of Colorado School of Medicine, Denver, CO

* Corresponding Author: John.reichard@UC.edu

Supplementary Figures

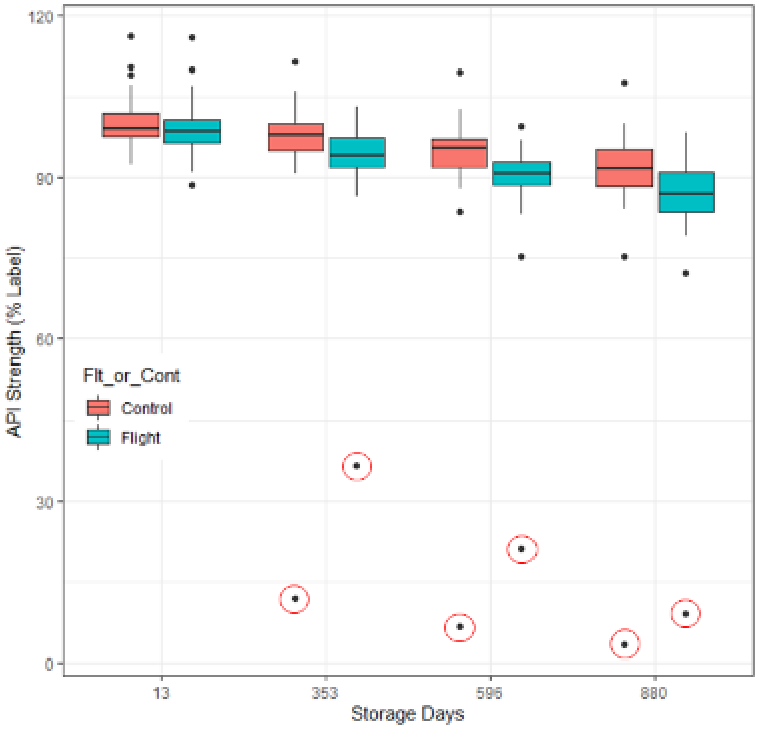

Supplementary Figure 1. Boxplot showing the distribution of all drug strengths reported by Du et al.(2011). Boxes represent the 25th to 75th interquartile ranges (IQR); whisks indicate the minimum and maximum values or ≤ 1.5 * IQR (inter quartile range). The median for each group is indicated by the bar within the IQR. Dots are observations that contain API outside 1.5*IQR. The dots circled in red are clavulanate, which was excluded from our analyses as an extreme outlier displaying increased stability with spaceflight storage compared to the lot-matched terrestrial control samples.

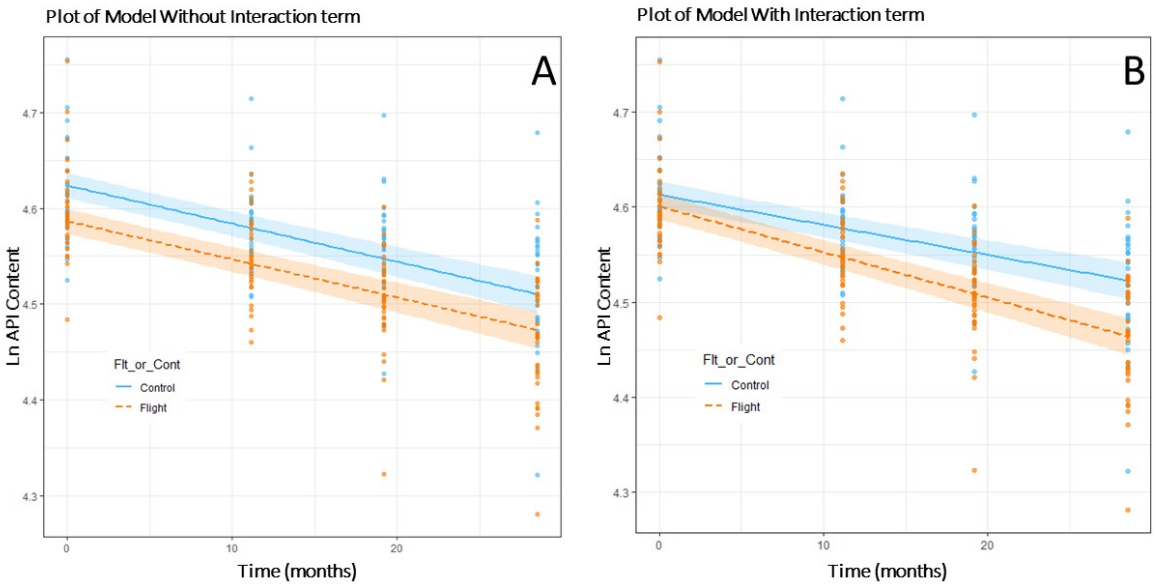

Supplementary Figure 2. Interaction plots. Panel A includes a variable for treatment group (terrestrial control or spaceflight) but no term for the interaction of storage condition with time. The interaction plot in panel B includes an interaction term. As a result, there is a clear change in slope, which is a positive indicator of an interaction between model variables.

Supplementary Tables

Supplementary Table 1. Summary data for Du *et al*. 2011 with one-way statistical significance for each lot-matched sample

Drug Name	Storage Days	Control API (% label)	Control Std (%)	Flight API (% label)	Flight Std. (%)	n	One-way p-Vale	BH Adj. p-Value	Lower C.I.	S
acyclovir	13	107.2	0.31	104.7	0.06	3	0.002	0.004	1.9921	*
amoxicillin	13	116.2	0.35	116	0.14	3	0.2173	0.245	-0.3435	N
clavulanate	13	93.3	0.59	91	0.79	3	0.0091	0.014	1.0578	
atorvastatin	13	101.1	0.66	100.2	0.88	3	0.1173	0.14	-0.485	N
azithromycin	13	109	0.81	106.9	0.62	3	0.0131	0.019	0.8195	
cefadroxil	13	99.3	2.24	102.3	1.33	3	0.9336	0.99	-6.4296	N
ciprofloxacin	13	104.8	0.87	101.5	0.31	3	0.0071	0.011	1.9358	
dextroamphetamine	13	98.1	0.83	98.3	0.6	3	0.6231	0.671	-1.497	N
fluconazole	13	96.3	0.51	96	0.27	3	0.2167	0.245	-0.4799	N
furosemide	13	99.1	0.71	93.9	0.54	3	0.0004	0.001	4.0792	*
Ibuprofen	13	98.4	0.84	100.1	0.05	3	0.9639	1	-3.1118	N
imipenem_(p)	13	97.6	1.43	103.4	0.88	3	0.9965	1	-7.9933	N
cilastatin_(p)	13	107	0.4	98.5	2.02	3	0.0077	0.012	5.1944	
levofloxacin	13	98.2	0.16	97.5	0.06	3	0.0048	0.08	0.4502	*

Levothyroxine	13	92.3	0.66	88.6	0.51	3	0.001	0.003	2.6543	*
Metoprolol	13	101.9	1.3	98	0.51	3	0.0116	0.017	1.878	
Metronidazole	13	97.5	0.42	96	0.28	3	0.0049	0.008	0.8513	*
Norethindrone	13	101.3	0.74	103.4	1.8	3	0.9149	0.978	-4.8893	N
ethinyl estradiol	13	94.3	0.87	96.8	0.68	3	0.9904	1	-3.8822	N
Phenytoin	13	100.9	0.51	98.5	0.84	3	0.01	0.015	1.1126	
Promethazine	13	95.6	0.21	95.4	0.31	3	0.2069	0.237	-0.2797	N
Risedronate	13	96.4	0.13	94.6	0.23	3	0.0005	0.001	1.4483	*
Sertraline	13	104.8	0.09	100.8	0.23	3	0.0001	0	3.6422	*
sulfamethoxazole	13	101	0.01	98.9	0.54	3	0.0107	0.016	1.1899	
Trimethoprim	13	102	0.1	99.6	0.64	3	0.0104	0.016	1.3418	
Temazepam	13	98.9	1.35	98.8	0.21	3	0.4551	0.494	-2.1324	N
ciprofloxacin_(o)	13	95.4	0.96	94.7	0.92	3	0.2068	0.237	-0.9374	N
clotrimazole_(c)	13	98.7	0.34	98.4	0.16	3	0.1326	0.157	-0.2221	N
mupirocin_(o)	13	98.2	0.16	99	0.42	3	0.9669	1	-1.4548	N
promethazine_(pr)	13	99.3	0.15	97.7	0.16	3	0.0001	0	1.3297	*
silver sulfadiazine_(c)	13	97.5	1.33	96	1	3		0.121	-0.5943	N
triamcinolone_(c)	13	98.6	0.59	98.3	0.54	3	0.2758	0.304	-0.6866	N
ciprofloxacin_(s)	13	98.7	0.4	96.5	0.24	3	0.0014	0.003	1.5873	*
epinephrine_(i)	13	110.5	0.23	110	0.21	3	0.0251	0.034	0.1158	
lidocaine_(i)	13	100.4	0.72	99.6	0.59	3	0.1067	0.129	-0.3586	N
promethazine_(i)	13	1035	1.8	100.9	0.17	3	0.0641	0.08	-0.4119	N
Acyclovir	353	106	0.29	101.5	0.1	3	0.0002	0.001	4.044	*
Amoxicillin	353	100.1	0.05	102.3	0.33	3	0.9968	1	-2.7462	N
Clavulanate	353	12	0.43	36.7	0.33	3	1	1	-25.3802	N
Atorvastatin	353	100.7	0.95	97.8	0.65	3	0.0079	0.012	1.4281	
Azithromycin	353	98.9	0.33	98.2	0.1	3	0.0282	0.038	0.1754	
Cefadroxil	353	99.2	0.28	93.5	0.53	3	0.0002	0.001	4.8895	*
Ciprofloxacin	353	92.9	0.15	92.1	1.19	3	0.1821	0.212	-1.1802	N
dextroamphetamine	353	98.3	1.07	89.5	1.09	3	0.0003	0.001	6.9198	*
Fluconazole	353	96	0.09	94.2	0.39	3	0.006	0.01	1.1674	
Furosemide	353	93.6	0.42	89.9	0.42	3	0.0002	0.001	2.9689	*
Ibuprofen	353	100.2	0.09	100	0.45	3	0.262	0.293	-0.536	N
imipenem_(p)	353	103	0.73	100.5	1.31	3	0.03	0.04	0.4971	
cilastatin_(p)	353	111.5	0.56	103.1	0.75	3	0.0001	0	7.2207	*
Levofloxacin	353	98	0.28	94.7	0.35	3	0.0001	0	2.7406	*
Levothyroxine	353	90.7	0.8	86.5	0.13	3	0.0052	0.009	2.8792	*
Metoprolol	353	95.3	0.2	88.9	0.56	3	0.0005	0.001	5.5221	*
Metronidazole	353	95.6	0.31	94.4	0.46	3	0.0126	0.018	0.4887	
Norethindrone	353	99.1	0.77	97.8	0.4	3	0.0403	0.052	0.122	N
ethinyl estradiol	353	98.4	0.81	97.4	0.63	3	0.0855	0.106	-0.2853	N
Phenytoin	353	8.5	0.31	96.4	0.3	3	0.0005	0.001	1.5689	*
Promethazine	353	93.2	0.22	91.6	0.17	3	0.0004	0.001	1.2514	*
Risedronate	353	94.9	0.56	93	0.5	3	0.0061	0.01	0.9726	
Sertraline	353	100.3	0.54	91.7	0.45	3	0	0	7.7267	*

Sulfamethoxazole	353	100	0.3	96.9	0.26	3	0.0001	0	2.6085	*
Trimethoprim	353	97.9	0.14	94.7	0.13	3	0	0	2.9645	*
Temazepam	353	90.9	0.38	87.6	0.08	3	0.0016	0.004	2.6802	*
ciprofloxacin_(o)	353	NA	NA	NA	NA	3	NA	NA	NA	N
clotrimazole_(c)	353	96.4	0.15	95.2	0.26	3	0.0025	0.005	0.8023	*
mupirocin_(o)	353	NA	NA	NA	NA	3	NA	NA	NA	N
promethazine_(pr)	353	97.3	0.4	92.4	0.1	3	0.0007	0.002	4.2547	
silver sulfadiazine_(c)	353	93.5	0.28	92.6	0.51	3	0.0362	0.047	0.1203	
triamcinolone_(c)	353	97.9	1.12	93.8	0.94	3	0.0045	0.008	2.2846	*
ciprofloxacin_(s)	353	NA	NA	NA	NA	3	NA	NA	NA	N
epinephrine_(i)	353	100.3	0.3	94	0.1	3	0.0001	0	5.8269	*
lidocaine_(i)	353	NA	NA	NA	NA	3	NA	NA	NA	N
promethazine_(i)	353	95.1	0.08	93.5	0.31	3	0.0044	0.008	1.101	*
Acyclovir	596	102.3	0.44	96.9	0.44	3	0.0001	0	4.6341	*
Amoxicillin	596	97.2	0.37	88	0.68	3	0.0001	0	8.1604	*
Clavulanate	596	6.6	0.16	21.1	1.1	3	0.9992	1	-16.3231	N
Atorvastatin	596	99.5	0.81	93.9	0.33	3	0.0014	0.003	4.3439	*
Azithromycin	596	95.6	0.48	92.2	0.55	3	0.0007	0.002	2.4968	*
Cefadroxil	596	97.2	0.16	92.8	0.14	3	0	0	4.137	*
Ciprofloxacin	596	91.5	0.03	90.6	0.34	3	0.0217	0.03	0.3306	
Dextroamphetamine	596	97.5	0.14	88.8	1.46	3	0.0044	0.008	6.2575	*
Fluconazole	596	91.7	0.4	90.2	0.69	3	0.0214	0.03	0.4444	
Furosemide	596	87.9	0.16	83.2	0.11	3	0	0	4.4519	*
Ibuprofen	596	98.8	0.26	95.9	0.54	3	0.0021	0.004	2.0717	*
imipenem_(p)	596	102.6	0.31	92.8	0.22	3	0	0	9.3171	*
cilastatin_(p)	596	109.6	0.5	99.6	0.25	3	0	0	9.2342	*
Levofloxacin	596	87.9	0.73	84.8	0.24	3	0.0058	0.01	1.9474	
Levothyroxine	596	83.7	0.78	75.4	0.29	3	0.0005	0.001	7.0814	*
Metoprolol	596	92.1	0.38	87.6	0.26	3	0.0001	0	3.9112	*
Metronidazole	596	93.9	0.22	92	0.46	3	0.0043	0.008	1.1941	*
Norethindrone	596	95.7	0.44	94.6	2.59	3	0.27	0.3	-3.1685	N
ethinyl estradiol	596	90.9	1.46	90.5	1.87	3	0.3928	0.43	-2.5701	N
Phenytoin	596	95.3	0.57	92.2	0.41	3	0.0011	0.003	2.2101	*
Promethazine	596	93.2	0.17	88.7	0.03	3	0.0002	0.001	4.2203	*
Risedronate	596	93.3	0.23	88.2	0.39	3	0.0001	0	4.503	*
Sertraline	596	96	0.61	88.1	0.63	3	0	0	6.8203	*
Sulfamethoxazole	596	96.8	0.1	90.9	0.06	3	0	0	5.7468	*
Trimethoprim	596	96.4	0.36	90.7	0.72	3	0.0006	0.002	4.5973	*
Temazepam	596	88.1	0.67	85.4	0.2	3	0.007	0.011	1.6336	
ciprofloxacin_(o)	596	93.2	0.62	90.3	0.27	3	0.0035	0.007	1.9433	*
clotrimazole_(c)	596	95.3	0.38	93.1	0.93	3	0.0199	0.028	0.7582	
mupirocin_(o)	596	94.3	0.24	89.3	0.36	3	0.0001	0	4.444	*
promethazine_(pr)	596	95.7	0.62	89.7	0.41	3	0.0002	0.001	5.0433	*
silver sulfadiazine_(c)	596	90.5	0.36	89.8	0.61	3	0.0893	0.11	-0.2337	N
triamcinolone_(c)	596	96.4	0.83	93.1	0.02	3	0.0102	0.016	1.9014	

ciprofloxacin_(s)	596	98.2	0.67	93.6	0.77	3	0.0008	0.002	3.3368	*
epinephrine_(i)	596	96.5	0.75	90.8	1.14	3	0.0017	0.004	3.9419	*
lidocaine_(i)	596	98.7	1.14	95.6	1.68	3	0.0327	0.043	0.4998	
promethazine_(i)	596	92.9	0.83	91.5	0.22	3	0.0457	0.058	0.0665	N
Acyclovir	880	100.1	0.53	92.4	0.94	3	0.0004	0.001	6.2627	*
Amoxicillin	880	96.4	0.47	87	0.41	3	0	0	8.6283	*
Clavulanate	880	3.3	0.31	9.1	2.07	3	0.9813	1	-9.2275	N
Atorvastatin	880	98.9	0.53	92.5	0.54	3	0.0001	0	5.4686	*
Azithromycin	880	94.8	0.39	90.9	0.37	3	0.0001	0	3.2378	*
Cefadroxil	880	95.6	0.45	92.1	0.07	3	0.0023	0.005	2.7559	*
Ciprofloxacin	880	90.9	0.3	84.4	0.49	3	0.0001	0	5.7487	*
Dextroamphetamine	880	87.3	0.03	84.5	0.34	3	0.0023	0.005	2.2306	*
Fluconazole	880	89.9	0.84	88.2	0.6	3	0.026	0.035	0.3901	
Furosemide	880	86.2	0.63	80.2	0.69	3	0.0002	0.001	4.8473	*
Ibuprofen	880	97.6	0.22	92.1	0.02	3	0.0002	0.001	5.1317	*
imipenem_(p)	880	96	0.75	90.6	0.24	3	0.0017	0.004	4.2131	*
cilastatin_(p)	880	107.7	0.96	98.3	0.11	3	0.0016	0.004	7.7991	*
Levofloxacin	880	85.6	0.89	81.2	0.31	3	0.0038	0.007	3.0017	*
Levothyroxine	880	75.3	0.14	72.3	0.3	3	0.0004	0.001	2.5391	*
Metoprolol	880	91.7	0.93	86.9	0.88	3	0.0015	0.004	3.2227	*
Metronidazole	880	93.3	0.49	90	0.85	3	0.0042	0.008	1.9998	*
Norethindrone	880	94.9	0.22	91	1.44	3	0.0199	0.028	1.5173	
ethinyl estradiol	880	88.7	1.18	86.9	0.31	3	0.055	0.069	-0.097	N
Phenytoin	880	93.8	0.42	90.4	0.29	3	0.0003	0.001	2.7484	*
Promethazine	880	87.5	0.37	83.7	0.26	3	0.0001	0	3.2245	*
Risedronate	880	91.7	0.01	86.5	0.45	3	0.0012	0.003	4.4417	*
Sertraline	880	95.6	0.23	86.9	0.45	3	0	0	8.0113	*
Sulfamethoxazole	880	90.3	0.91	80.7	0.64	3	0.0001	0	8.1844	*
Trimethoprim	880	88.8	1.45	79.1	0.51	3	0.0018	0.004	7.4238	*
Temazepam	880	84.2	0.14	80.8	0.91	3	0.0105	0.016	1.8946	
ciprofloxacin_(o)	880	88.5	0.47	83.4	0.46	3	0.0001	0	4.2904	*
clotrimazole_(c)	880	90.3	0.31	87.3	0.61	3	0.0025	0.005	2.0663	*
mupirocin_(o)	880	87	0.56	82.9	0.14	3	0.0021	0.004	3.1965	*
promethazine_(pr)	880	93.9	0.54	84	0.76	3	0.0001	0	8.7159	*
silver sulfadiazine_(c)	880	88.3	0.15	83.9	0.05	3	0.0001	0	4.1634	*
triamcinolone_(c)	880	94.8	0.31	92.3	0.36	3	0.0004	0.001	1.9115	*
ciprofloxacin_(s)	880	96.2	0.46	91.6	0.77	3	0.0011	0.003	3.4208	*
epinephrine_(i)	880	91.7	0.6	87.2	0.5	3	0.0003	0.001	3.5296	*
lidocaine_(i)	880	95.3	1.18	94	1.45	3	0.1487	0.175	-1.0285	N
promethazine_(i)	880	91.6	0.36	87.1	0.62	3	0.0006	0.002	3.5514	*

Abbreviations: API, Active pharmaceutical ingredient; Std, Standard deviation; n, sample number; BH, Benjamini-Hochberg adjustment for multiple comparisons, C.I., Confidence interval, N.S., BH-adjusted p-values not significant ($p \geq 0.05$), * BH-adjust p-value significant at $p < 0.05$; **, BH-adjusted p-values highly significant at $p < 0.01$.

**Supplementary Table 2.** Difference[1] in API potency (%) between spaceflight and terrestrial control drugs					
API Name	Formulation	Timepoint			
		13	353	596	880
Acyclovir	Tablet	2.5	4.5	5.4	7.7
Amoxicillin	Tablet	0.2	-2.2	9.2	9.4
Atorvastatin	Tablet	0.9	2.9	5.6	6.4
Azithromycin	Tablet	2.1	0.7	3.4	3.9
Cefadroxil	Capsule	-3.0	5.7	4.4	3.5
Cilastatin	Powder	8.5	8.4	10	9.4
Ciprofloxacin	Ointment	0.7	NA	2.9	5.1
Ciprofloxacin	Solution	2.2	NA	4.6	4.6
Ciprofloxacin	Tablet	3.3	0.8	0.9	6.5
Clotrimazole	Cream	0.3	1.2	2.2	3.0
Dextroamphetamine	Tablet	-0.2	8.8	8.7	2.8
Epinephrine	Injection	0.5	6.3	5.7	4.5
Fluconazole	Tablet	0.3	1.8	1.5	1.7
Furosemide	Tablet	5.2	3.7	4.7	6.0
Ibuprofen	Tablet	-1.7	0.2	2.9	5.5
Imipenem	Powder	-5.8	2.5	9.8	5.4
Levofloxacin	Tablet	0.7	3.3	3.1	4.4
Levothyroxine	Tablet	3.7	4.2	8.3	3
Metoprolol	Tablet	3.9	6.4	4.5	4.8
Metronidazole	Tablet	1.5	1.2	1.9	3.3
Mupirocin	Ointment	-0.8	NA	5.0	4.1
Norethindrone	Tablet	-2.1	1.3	1.1	3.9
Phenytoin	Capsule	2.4	2.1	3.1	3.4
Promethazine[2]	Injection	2.6	1.6	1.4	4.5
Promethazine[2]	Suppository	1.6	4.9	6.0	9.9
Promethazine	Tablet	0.2	1.6	4.5	3.8
Risedronate	Tablet	1.8	1.9	5.1	5.2
Sertraline	Tablet	4.0	8.6	7.9	8.7
Ag_sulfadiazine	Cream	1.5	0.9	0.7	4.4
Sulfamethoxazole	Tablet	2.1	3.1	5.9	9.6
Temazepam	Capsule	0.1	3.3	2.7	3.4
Triamcinolone	Cream	0.3	4.1	3.3	2.5
Trimethoprim	Tablet	2.4	3.2	5.7	9.7

[1] Drug products where API content was significantly ($p < 0.05$) less in spaceflight samples than the corresponding lot-matched controls in a one-tailed t-test within the total timeframe of the experiment (i.e., space-exposed samples were more degraded)

[2.] In the Du *et al.*,2011 publication, panels b and c of Figure 4 (promethazine) are switched based on the accompanying published supplementary data (Du et al. 2011).

NA = not available

API	Control Degradation Rate (%/day)	Spaceflight Degradation Rate (%/day)	**Rate Ratio (flight/ control)**	Estimated Control Half-life (Years)	Estimated Spaceflight Half-life (Years)	Control, API Percent remaining after 3 years	Spaceflight, API Percent remaining after 3 years
imipenem_(p)#	-1.73E-05	-1.63E-04	**9.43**	109.8	11.65	98.12	83.65
ibuprofen	-1.56E-05	-1.10E-04	**7.02**	121.53	17.32	98.30	88.68
ciprofloxacin_(s) #	-1.97E-05	-5.68E-05	**2.89**	96.57	33.45	97.87	93.97
atorvastatin	-3.50E-05	-9.80E-05	**2.8**	54.22	19.37	96.24	89.82
cefadroxil	-4.80E-05	-1.23E-04	**2.57**	39.56	15.42	94.88	87.38
promethazine_(pr) #	-7.00E-05	-1.68E-04	**2.41**	27.11	11.27	92.62	83.15
risedronate	-5.64E-05	-1.11E-04	**1.97**	33.68	17.10	94.01	88.55
acyclovir	-7.27E-05	-1.43E-04	**1.96**	26.13	13.30	92.35	85.53
trimethoprim	-1.40E-04	-2.55E-04	**1.81**	13.52	7.46	85.74	75.68
norethindrone#	-8.70E-05	-1.48E-04	**1.71**	21.82	12.79	90.91	85.00
sulfamethoxazole	-1.38E-04	-2.28E-04	**1.65**	13.76	8.33	85.97	77.90
ciprofloxacin_(o) #	-8.63E-05	-1.40E-04	**1.63**	22.01	13.52	90.99	85.74
amoxicillin	-2.17E-04	-3.47E-04	**1.60**	8.77	5.46	78.88	68.35
mupirocin_(o) #	-1.28E-04	-2.05E-04	**1.60**	14.82	9.27	86.91	79.91
Ethinyl estradiol#	-8.59E-05	-1.36E-04	**1.59**	22.11	13.93	91.02	86.13
promethazine	-9.51E-05	-1.47E-04	**1.55**	19.97	12.91	90.11	85.12
sertraline	-1.12E-04	-1.75E-04	**1.55**	16.90	10.88	88.42	82.61
triamcinolone_(c) #	-4.54E-05	-6.88E-05	**1.52**	41.86	27.62	95.15	92.75
metronidazole	-4.94E-05	-7.27E-05	**1.47**	38.47	26.13	94.74	92.35
clotrimazole_(c) #	-9.77E-05	-1.36E-04	**1.39**	19.43	13.95	89.85	86.16
phenytoin	-8.37E-05	-1.11E-04	**1.33**	22.69	17.10	91.24	88.55
dextroamphetamine	-1.27E-04	-1.62E-04	**1.27**	14.90	11.73	86.98	83.75
lidocaine_(i) #	-5.42E-05	-6.91E-05	**1.27**	35.02	27.47	94.23	92.71
ciprofloxacin	-1.56E-04	-1.96E-04	**1.26**	12.16	9.68	84.28	80.66
levofloxacin	-1.79E-04	-2.25E-04	**1.26**	10.63	8.45	82.23	78.19
Ag_sulfadiazine_(c) #	-1.16E-04	-1.46E-04	**1.26**	16.36	12.97	88.06	85.18
temazepam	-1.81E-04	-2.22E-04	**1.23**	10.50	8.57	82.03	78.45
azithromycin	-1.62E-04	-1.94E-04	**1.2**	11.76	9.78	83.79	80.85
epinephrine_(i) #	-2.17E-04	-2.59E-04	**1.19**	8.77	7.34	78.89	75.33
fluconazole	-8.63E-05	-1.00E-04	**1.16**	21.99	18.99	90.98	89.63
furosemide	-1.69E-04	-1.95E-04	**1.15**	11.25	9.75	83.13	80.80
promethazine_(i) #	-1.37E-04	-1.56E-04	**1.14**	13.88	12.16	86.08	84.28
metoprolol	-1.21E-04	-1.37E-04	**1.13**	15.73	13.88	87.61	86.08
levothyroxine	-2.45E-04	-2.55E-04	**1.04**	7.75	7.46	76.46	75.66
clavulanate	-3.80E-03	-2.62E-03	**0.69**	0.50	0.72	1.56	5.66
cilastatin_(p) #	9.73E-06	-9.89E-06	**-1.02**	-195.14	191.98	101.07	98.92

Supplementary Table 3. Degradation rate estimates and shelf-life predictions for exploratory space missions

Abbreviations: o = ointment, c=cream, s = suppository, i = injectable solution, p = powder, pr = suppository, NA = not applicable, Ag = Silver.

*The least-squared regression model for Cilastatin exhibited no degradation resulting in a negative value.

Medications that were *not* repackaged

Supplementary Table 4. GEE estimated marginal means for the response

Storage days	Terrestrial Predicted	Terrestrial 95% CI	Spaceflight predicted	Spaceflight 95% CI
13	100.77	99.47, 102.08	99.42	98.05, 100.82
353	97.26	95.96, 98.58	94.25	93.02, 95.50
596	94.83	93.36, 96.33	90.72	89.36, 92.10
880	92.07	90.29, 93.89	86.76	85.10, 88.45

Supplementary Table 5. Summary of Drug Products Tested in Opportunistic Studies

Drug product	Manufacturer	Studies	Flight days	Percent label strength	Day past expiration date at testing	Significance Level (p-value)[1]
Amoxicillin (cap) 500 mg	Sandoz	(Cory 2017)	0<br>356<br>498<br>501	115.9 ± 1.8<br>114.1 ± 1.5<br>114.3 ± 1.4<br>112.4 ± 1.1	Not Exp.<br>28<br>18<br>8	0.740
Azithromycin (tab) 250 mg	NA	(Wu and Chow 2016)	NA	100.0 0.8<br>114.0 ± 1.1<br>109.2 ± 1.1	NA	0.540
	Greenstone	(Khan and Wotring 2014)	316	98.3 ± 0.9	366	
	Sandoz		880	98.3 ± 2.0	1355	
Ibuprofen (tab) 400 mg	NA	(Wu and Chow 2016)	0<br>551<br>647<br>140	100.0 ± 0.6<br>82.7 ± 1.0<br>85.8 ± 2.0<br>85.2 ± 2.2	NA	0.188
	NA	(Wotring 2016)	550	99.9 ± 1.8	NA	
	Dr. Reddy's Lab	(Cory 2016)	0<br>140	100.1 ± 0.9<br>99.8 ± 0.8	12<br>Not exp.	
Levofloxacin (tab) 500 mg	Sandoz	(Cory 2016)	132<br>0	99.7 ± 1.4<br>98.8 ± 1.5	Not exp.	0.656
	Janssen	(Khan and Wotring 2014)	316<br>880	104.2 ± 0.22<br>101.1 ± 0.61	Not exp<br>1264	
Levothyroxine (tab) 0.025 mg	Sandoz	(Khan and Wotring 2014)	880	81.39 ± 1.47	1690	0.30344
Phenytoin 300 mg	Mylan	(Cory 2016)	0 (control)<br>241	101.5 ± 0.7102.2<br>± 0.5	12<br>8	0.28103
Promethazine (tab) 250 mg	NA	(Wu and Chow 2016)	0<br>256<br>193<br>432	100.0 ± 1.0 NA<br>76.5 ± 1.3 NA<br>79.5 ± 1.3 NA<br>86.4 ± 1.1 NA	NA	0.174
Promethazine (inj) 25 mg/mL	Mylan	(Wu and Chow 2016)	0<br>256<br>565	100.0 ± 1.1<br>81.5 ± 1.6 NA<br>84.6 ± 1.7	NA	0.1294
Sertraline (tab) 50 mg	Greenstone	(Cory 2016)	0<br>700	99.5 ± 5.2<br>98.6 ± 5.8	Not exp.<br>43	0.0647

1. Significance is Flight versus control in the combined data regression model.
NA = not available, Not exp. = not expired, cap = capsule, tab = tablet, inj = injectable.

Supplementary Methods

Literature search and study design:

To help assure that the information supporting our analysis was comprehensive, a literature search was performed to identify any available English language studies with empirical spaceflight drug stability data. A Boolean search of NCBI Medline, Embase and Web of Science was performed using MeSH/keyword search strings including "spaceflight OR spacecraft", "stability OR 'drug stability' OR degradation", "drug OR pharmaceutical OR medication", "storage OR shelf-life." Additionally, a tree-search strategy ad Google search identified unpublished NASA reports. The rule-out criteria included spaceflight analog studies, studies of nutritional supplements, and reviews that republished previously reported results from primary studies.

Degradation kinetics

Loss of drug potency was evaluated based on both zero-order and first-order reaction kinetics. For the available data, either kinetic model is appropriate because, when the degradation rate is slow and the extent of degradation exceeds ~50% of the label strength the total, then zero, first and second-order kinetics yield very similar fits and predictions (Altan and Raghavarao 2003). Thus, both zero- and first-order degradation kinetics were evaluated, and the most parsimonious model was selected. Typically, model selection is based on metrics such as R-square and Akaike information criterion (AIC); however, these methods are only appropriate when input data for the different models are the same. Here, the input data are either arithmetic (zero-order) or log-transformed (first-order) API concentrations; therefore, the preferred model was selected using a standard log-likelihood procedure together with an adjustment to compensate for the log transformation.

A first-order rate model is used to estimate API half-life from regression slope coefficient (k) with an adjustment converting storage days to years, using the formula for first-order kinetics as shown in Supplementary Equation 1,

$$(1) \qquad Half-life\ (years) = \frac{\ln(2)}{slope\ (days^{-1}) \times \left(\frac{365.24\ days}{1\ year}\right)}$$

Determination of slope derived from the exponentiated β coefficient of the regression line calculated from the natural log of the percent strength reported for each drug as a function of storage time.

FDA and European Medicines Agency (EMA) guidance documents assume that drug degradation during long-term storage follows linear kinetics when represented by an arithmetic function or a semi-logarithmic scale (US Food and Drug Administration.(ICH 2003; US FDA 2004) Therefore, the suggested statistical approach for analyzing manufacturing lot and storage condition data is a regression model (least-squares method). Regression models can test the null hypothesis of equality of slope or intercept relative to a control sample. The intercept is the amount of active substance (or other selected drug attributes) at the start of the test (i.e., time = 0). The regression coefficient(s) and the lower 95% confidence interval describe the degradation rate as a function of time and are used to predict the retest period and shelf life (Almalik et al. 2014; ICH 2003; Kirkwood 1977).

Supplementary Table 6provided the mean likelihood results for each regression model (linear and ln-linear) for both control and spaceflight samples. The likelihood was determined in R (Boston, MA) using the "distributions3" package (version 0.1.1).The Log-likelihood evaluation showed a minimal difference in the overall fit for the two models, with the first-order (ln-linear) having a slightly more positive likelihood estimate. Due to the practical considerations that included consistency with planned degradation experiments, general applicability to either zero- or first-order reactions when rates are low (exemplified by the degradation kinetics of clavulanate in Supplementary Figure 1), and limitations of zero-order models when applied to percent-of-label analyses (Altan and Raghavarao 2003), a first-order kinetic model was selected for evaluation throughout the following analysis.

Supplementary Table 6. Mean Log-likelihood for regression model comparisons

Regression model	Mean Likelihood*	Std
Linear, Control	-10.71097	2.850591
Ln-Linear, Control	-10.58913	2.508225
Linear, Flight	-12.15926	2.078280
Ln-Linear, Flight	-12.10451	1.950584
*higher likelihoods are more probable		

Statistical analysis

All statistical analyses were performed using open-source R statistical software (version 4.0.5) using R Studio (Boston, MA version 1.4.1106) as described in the Supplemental Methods section.

The t-test was performed to test the hypothesis that medications exposed to spaceflight have less API content compared to lot-matched terrestrial control samples at each time point under the assumption of normality and homoscedasticity. A one-sided t-test was used because the focus of this study is only on whether or not drugs exposed to spaceflight are *less stable* than terrestrial controls. Loss of API content constitutes a medical risk for therapeutic failure and exposure to potentially hazardous impurities. A one-sided t-test ignores the alternative possibility that the API might be *more* stable during spaceflight, which is appropriate because prolonged API stability does not contribute to therapeutic failure. For the sake of completeness, one-sided t-tests were performed using the tsum.test () function from the BSDA R statistical package (version 1.2.0), which takes as input summary statistics (mean, standard deviation, and n) for each lot-matched pair of samples tested. Benjamini & Hochberg (1995) correction was applied to adjust for multiple comparisons using the p.adjust () function of the R stats package (version 4.0.5) (Supplementary Table 1). All other t-tests for grouped samples (i.e., samples stored for 13 days vs. 880 days) used the t-test function from the stats R package, generally without correction for multiple comparisons.

Linear regression of API content was performed in R studio using natural log-transformed API content using the R software lm() function. An effort was made to incorporate statistical variability for each data point into the regression models. However, the very low variability raised uncertainties regarding the independence of each replicate which justifies treating each mean value of replicate samples as single independent observations. Original raw data were located for all the remaining unpublished and published NASA reports.

A parametric Bayesian accelerated failure time (AFT) model, based on a Weibull distribution, was used in this analysis since AFT models so as not to be constrained by a constant (proportional) hazards assumption. The Weibull distribution was selected based on a visual inspection of plots of the semi-parametric baseline model to parametric models fit with different distributions, and compared against the Cox proportional hazard model and the maximum likelihood model. The parametric Weibull model is defined in terms of location (l) and scale (σ) parameters. In the absence of previous studies on the effect of spaceflight on drug stability, a flat ("uninformed") prior is used to allow the data to drive the estimation more than any particular prior assumptions. The Weibull ATF model is formulated as shown in Supplementary Equation 2,

$$(2) \qquad \log(t_i) = \beta_0 + \beta_1 x_{flight,i} + \sigma \in_i \qquad i = 1, 2, \ldots, n$$

where β_0 and β_1 are regression coefficients for the intercept and spaceflight conditions, respectively, subscript i corresponds to each API, σ is the Weibull scale parameter, and ϵ is a model error. Bayesian parametric survival analysis was performed in R with the "icenreg" (version 2.0.15) package supported by the "survival" (3.2-10) and "survminer" (version 0.4.9) packages.

Mixed-model regression was performed in addition GEE estimation. Since each medication contains a different API and requires different processing for analytical analysis, it is reasonable to assume that each different drug formulation is a distinct and independent entity. Therefore, each drug product is independent test. However, over the duration of the experiment, repeated measurements of API strength for each drug are not independent, since these are serial measurements of the same drug over time. These repeated measures are accounted for by using a mixed effect model that incorporates drug-specific random effects, both intercepts and slopes. Mixed model analysis was performed using the “nlme” and “lme4” packages in R with the lme() and lme4() functions, respectively. Prior to data analysis, storage days were converted to months (365/12). Model selection was performed using the anova () function of the Stats R package based on AIC and BIC values of lmer4 models generated with attributes REML=FALSE for maximum likelihood (ML) comparison and lmerControl(optimizer = "Nelder_Mead") to improve model convergence.

Supplementary Note 1

The evaluation of terrestrial and spaceflight treatments is analogous to the evaluation of whether or not results from independently performed stability tests can be combined, under FDA shelf-life stability testing guidance (Capen et al. 2012; US FDA 2004). Linear mixed-effect regression models have been utilized as one approach for such drug stability hypothesis testing (Chen et al. 1995; van den Heuvel, E. R. et al. 2011). An advantage of mixed-model regression over conventional univariate or multivariate regression approaches, as recommended for shelf-stability analysis, is that by combining data and pooling standard error, mixed-effect regression increase the degrees of freedom and hence yields more precise parameter estimates and improved statistical inference (Almalik et al. 2014; Chen et al. 1995; van den Heuvel, E. R. et al. 2011). Here, we make the conservative assumption that different APIs, and potentially different drug formulations containing the same API (e.g. tablet, injectable), have different susceptibilities for degradation over time, and therefore a random slope factor is justified to account for variability in degradation rate (Supplementary Table 3). Hence, the postulated mixed model includes a fixed factor for storage time (in units of months of storage), a fixed factor for treatment group, a random intercept for

the variable baseline observed for each drug (random intercepts) and a random factor for repeated measurements of each API (random slopes).

In addition to the fixed and random effects, an interaction term is also evaluated for inclusion in the model to account for the combined effects of storage time with treatment group (flight vs. control). This interaction is mechanistically justified since the storage time cumulatively increases exposure to latent and observed environmental factors (temperature, humidity, CO_2, ionizing radiation) or a combination of factors that can contribute to API degradation. This interaction was evaluated by the interaction plot (Supplementary Figure 2) and based on minimizing the absolute Akaike and Bayesian information criterion (AIC and BIC, respectively) as well as maximizing the model significance by the ANOVA statistic (Supplementary Table 7). These tests support an apparent interaction between time and storage condition, which is incorporated into the model. The most parsimonious postulated model recognizes that each API has its own inherent degradation rate (slope coefficient) that is independently affected by storage conditions (i.e., control vs. flight), as well as the physicochemical properties of the drug formulation (*inclusive of* excipients).

Together, control and spaceflight drugs start out with an average of approximately 100.03% of the label amount of API. However, between the two storage conditions there is a statistically significant difference in the estimated mean strength at the outset of the experiment of 1.3% of label strength (Supplementary Table 8). At the 13-day timepoint, the model-predicted API strength is 100.5% label strength in controls and 99.1% in spaceflight samples. The difference in API content at the first early 13-day timepoint is not entirely attributable to time-dependent degradation; rather, the difference is partly attributable to differences in API amount at the initiation of the experiment. This difference is not directly attributable to the samples themselves, which are pair-matched from the same manufacturing lot, but could reflect differences in repackaging time if controls and spaceflight samples were prepared separately, or exposure to off-nominal storage conditions of flight samples prior to analysis.

Supplementary Table 7. Regression model formulations and model selection values.

Model	Model Formulation	npar	AIC	BIC	logLik	Pr(>Chisq)
Baseline linear model	$\ln(API\%) = \beta_{intercept} + (\beta_{Storage} \times Time_{Months}) + \varepsilon$					
	log(Pct_API) ~ Flight_days	3	-807	-797	406.7	NA
Linear multiple regression	$\ln(API\%) = \beta_{int} + (\beta_{Stor} \times Time_{Mos}) + (\beta_{flight} \times TxGrp) + \varepsilon$					
	log(Pct_API) ~ Flight_days + Flt_or_Cont	4	-840	-825	423.9	4.87E-09

Linear multiple regression with interaction term	$\ln(API\%) = \beta_{int} + (\beta_{Stor} \times Time_{Mos}) + (\beta_{flight} \times TxGrp) + (\beta_{intaction} \times Time_{Mo} \times TxGrp) + \varepsilon$					
	log(Pct_API) ~ Cen.StorMo * Flt_or_Cont	5	-846	-827	427.8	NA
Random intercept	$\ln(API\%) = \beta_{int} + (\beta_{Stor} \times Time_{Mos}) + (\beta_{flight} \times TxGrp) + b_{API,int} + \varepsilon$					
	log(Pct_API) ~ Stor_mo + Flt_or_Cont + (1 | API_Name)	5	-1008	-990	509	<2.20E-16
Random intercept with interaction term	$\ln(API\%) = \beta_{int} + (\beta_{Stor} \times Time_{Mos}) + (\beta_{flight} \times TxGrp) + (\beta_{intaction} \times Time_{Mos} \times TxGrp) + b_{API,int} + \varepsilon$					
	log(Pct_API) ~ Stor_mo * Flt_or_Cont + (1 | API_Name)	6	-1027	-1005	519.5	4.66E-06
Mixed model	$\ln(API\%) = \beta_{int} + (\beta_{Stor} \times Time_{Mos}) + (\beta_{treat} \times TxGrp) + b_{API,int} + b_{API,stor} + \varepsilon$					
	log(Pct_API) ~ Stor_mo + Flt_or_Cont + (Stor_mo | API_Name)	7	-1066	-1040	539.8	1.82E-10
**Mixed model with interaction term***	$\ln(API\%) = \beta_{int} + (\beta_{Stor} \times Time_{Mos}) + (\beta_{flight} \times TxGrp) + (\beta_{intaction} \times Time_{Mos} \times TxGrp) + b_{API,int} + b_{API,stor} + \varepsilon$					
	log(Pct_API) ~ (Stor_mo) * Flt_or_Cont + (Stor_mo | API_Name)	8	-1099	-1070	557.4	3.06E-09

* Final model
Abbreviations: API = active pharmaceutical ingredient; Pct_API = Percent of label API strength; Int. = intercept; Stor = storage time; Mo = months; Txgrp = treatment group (flight vs. control); npar = Number of model parameters; AIC = Akaike information; BIC = Bayesian information criterion; LogLik = log-likelihood; Pr(>Chisq)=p-value

After controlling for differences in the intercepts and the degradation rate between different APIs, the first-order degradation rate for APIs under terrestrial conditions was -0.00318/month ($t_{½}$ = 218 months). This compares to a degradation rate of -0.00479/month ($t_{½}$ =145 months) for spaceflight samples, which equated to a 1.5-fold (51%) increase or an *additional* rate of-0.0016/month. The overall first-order degradation of all drugs stored terrestrially, compared to controls, is shown in Supplementary Table 3. Converted to an arithmetic scale, this equates to an additional ~0.2% of API degradation per month when averaged over the total duration of the experiment. As expected, variability among the different APIs contributes 71.3% of the overall variance (Supplementary Table 9), which represents the deviation of each API from the fixed effect intercept (β_0) value. This variability is likely to be primarily related to the starting percentage of API in each drug formulation. Typically, manufacturers aim to formulate drugs to 100% label strength at the time of manufacturing (US FDA 2004). The observed variability demonstrates that it should not be assumed that a drug's strength is 100% at the point that the manufacturer's container system is opened by the pharmacy or consumer. By comparison, the random slope captures the deviation of each API from the fixed effect slope (β_1); that is, the variance in degradation rate across all APIs under either terrestrial and spaceflight conditions (Barr et al. 2013). The random slope accounts for very little (<1%) of the model's total variance suggesting that among the

different APIs, degradation rate is relatively similar. Low correlation (-0.17) between the random intercept and slope suggests that there is no relationship between the degradation rate and the starting amount of API; that is, drugs that are more susceptible to degradation do not start out with either greater or lesser amounts of API. The remaining ~28% regression error was unexplained variance. Overall, the fixed-effect results align remarkably well with the GEE model results.

Supplementary Table 8. Linear mixed model regression fixed effect parameters[1]

Term	Coefficient	Standard Error	t-value	p-value
Intercept	4.61E+00	6.96E-03	663.151	< 2e-16
Storage (months)	-3.18E-03	3.44E-04	-9.225	2.83E-12
Treatment (Flight/Control)	-1.26E-02	4.86E-03	-2.586	0.0104
Interaction (Storage *flight)	-1.62E-03	2.62E-04	-6.196	3.18E-09

1. Model represents Ln(response) as shown in Supplementary Table 7 “Mixed model with interaction term”

Supplementary Table 9. Linear mixed model regression random effect parameters[1]

Group	Effect	Variance	Sd	Correlation	% of total variance
API	Intercept	1.30E-03	3.62E-02		71.34
API	Storage (Months)	2.97E-06	1.72E-03	-0.17	0.16
Residual	–	5.20E-04	2.28E-02		28.5
Total variance	–	1.82E-03			100

1. Model represents Ln(response) as shown in footnote Supplementary Table 7 “Mixed model with interaction term”

Supplementary References

Almalik O, Nijhuis MB, van den Heuvel, Edwin R (2014) Combined Statistical Analyses for Long-Term Stability Data with Multiple Storage Conditions: A Simulation Study 24:493-506. doi: 10.1080/10543406.2014.888439.

Altan S, Raghavarao D (2003) A Note on Kinetic Modeling of Stability Data and Implications on Pooling 13:425-430. doi: 10.1081/BIP-120022764.

Barr DJ, Levy R, Scheepers C, Tily HJ (2013) Random effects structure for confirmatory hypothesis testing: Keep it maximal 68:255-278. doi: 10.1016/j.jml.2012.11.001.

Capen R, Christopher D, Forenzo P, Ireland C, Liu O, Lyapustina S, O'Neill J, Patterson N, Quinlan M, Sandell D, Schwenke J, Stroup W, Tougas T (2012) On the shelf life of pharmaceutical products. AAPS PharmSciTech 13:911-918. doi: 10.1208/s12249-012-9815-2.

Chen JJ, Hwang J, Tsong Y (1995) Estimation of the shelf-life of drugs with mixed effects models 5:131-140. doi: 10.1080/10543409508835102.

Cory WC (2017) Analysis of Degradation of Pharmaceuticals Stored on the International Space Station - Final Report.

Cory WC (2016) Analysis of Degradation of Pharmaceuticals Stored on the International Space Station:1-250923-000193.

Du B, Daniels V, Vaksman Z, Boyd J, Crady C, Putcha L (2011) Evaluation of Physical and Chemical Changes in Pharmaceuticals Flown on Space Missions. AAPS J 13:299-308. doi: 10.1208/s12248-011-9270-0.

ICH (2003) Evaluation for Stability Data Q1E(S4)https://database.ich.org/sites/default/files/Q1E_Guideline.pdf.

Khan M, Wotring VE (2014) FDA Comprehensive Stability Evaluation of Three Medications :1-82.

Kirkwood TB (1977) Predicting the stability of biological standards and products. Biometrics 33:736-742.

US FDA (2004) Q1E Evaluation of Stability Datahttps://www.fda.gov/regulatory-information/search-fda-guidance-documents/q1e-evaluation-stability-data.

van den Heuvel, E. R., Almalik O, Nijhuis MB, Warner EI (2011) Statistical analysis for long-term stability studies with multiple storage conditions. Drug Inf J 45:301-314. doi: 10.1177/009286151104500310.

Wotring VE (2016) Chemical Potency and Degradation Products of Medications Stored Over 550 Earth Days at the International Space Station. AAPS J 18:210-216. doi: 10.1208/s12248-015-9834-5 [doi].

Wu L, Chow D (2016) Final report of NASA first award project.
